# TMED4 facilitates regulatory T cell suppressive function via ROS homeostasis in tumor and autoimmune mouse models

**DOI:** 10.1172/JCI179874

**Published:** 2024-10-31

**Authors:** Zhenyan Jiang, Huizi Wang, Xiaoxia Wang, Hongrui Duo, Yuexiao Tao, Jia Li, Xin Li, Jiamin Liu, Jun Ni, Emily Jiatong Wu, Hongrui Xiang, Chenyang Guan, Xinyu Wang, Kun Zhang, Peng Zhang, Zhaoyuan Hou, Yong Liu, Zhengting Wang, Bing Su, Bo Li, Youjin Hao, Bin Li, Xuefeng Wu

**Affiliations:** 1 Center for Immune-Related Diseases at Shanghai Institute of Immunology, Ruijin Hospital,; 2 Hongqiao International Institute of Medicine, Shanghai Tongren Hospital,; 3 Key Laboratory of Cell Differentiation and Apoptosis of the Chinese Ministry of Education, and; 4 Shanghai Institute of Immunology, Department of Immunology and Microbiology, Shanghai Jiao Tong University School of Medicine (SJTU-SM), Shanghai, China.; 5 Songjiang Research Institute, Songjiang District Central Hospital, Shanghai Jiao Tong University School of Medicine (SJTU-SM), Shanghai, China.; 6 Research Group of Computational and Integrative Biology, College of Life Sciences, Chongqing Normal University, Chongqing, China.; 7 Department of Pharmacology and Chemical Biology, Shanghai Key Laboratory of Emotions and Affective Disorders, SJTU-SM, Shanghai, China.; 8 Department of Biochemistry and Molecular Cell Biology, Shanghai Key Laboratory for Tumor Microenvironment and Inflammation, SJTU-SM, Shanghai, China.; 9 Hubei Key Laboratory of Cell Homeostasis, College of Life Sciences, Wuhan University, Wuhan, China.; 10 Department of Gastroenterology, Ruijin Hospital, SJTU-SM, Shanghai, China

**Keywords:** Immunology, Adaptive immunity, Autoimmune diseases, T cells

## Abstract

Endoplasmic reticulum stress (ERS) plays crucial roles in maintaining Treg stability and function, yet the underlying mechanism remains largely unexplored. Here, we demonstrate that (*Tmed4^ΔTreg^*) mice with Treg-specific KO of ERS-related protein transmembrane p24 trafficking protein 4 (TMED4) had more Tregs with impaired Foxp3 stability, Treg signatures, and suppressive activity, which led to T cell hyperactivation and an exacerbated inflammatory phenotype and boosted antitumor immunity in mice. Mechanistically, loss of *Tmed4* caused defects in ERS and a nuclear factor erythroid 2–related factor 2–related (NRF2-related) antioxidant response, which resulted in excessive ROS that reduced the Foxp3 stability and suppressive function of Tregs in an IRE1α/XBP1 axis–dependent manner. The abnormalities could be effectively rescued by the ROS scavenger, NRF2 inducer, or by forcible expression of IRE1α. Moreover, TMED4 suppressed IRE1α proteosome degradation via the ER-associated degradation (ERAD) system including the ER chaperone binding immunoglobulin protein (BIP). Our study reveals that TMED4 maintained the stability of Tregs and their suppressive function through IRE1α-dependent ROS and the NRF2-related antioxidant response.

## Abstract 2

ER stress responsive protein TMED4 maintains Treg function through BiP-IRE1α-XBP1 axis

## Graphical abstract

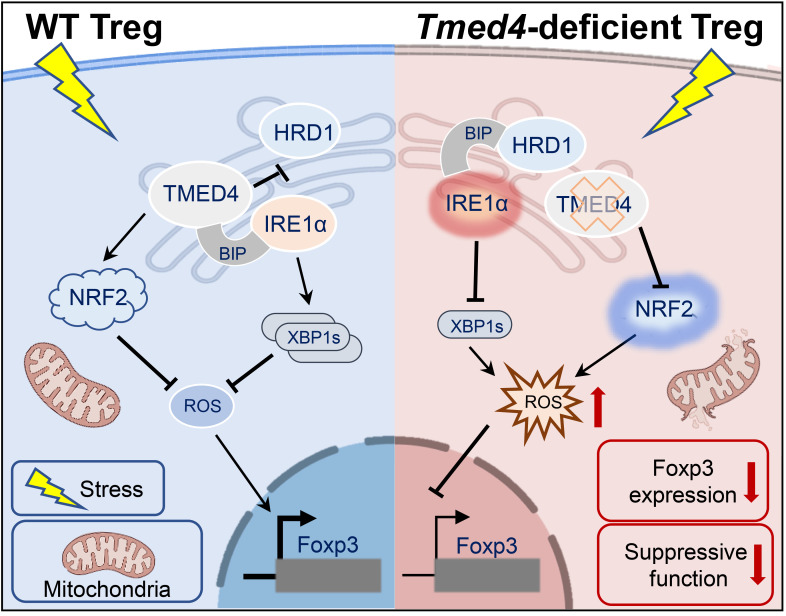

## Introduction

Tregs, a distinct subset of CD4^+^ T cells, perform an immunosuppressive function to maintain immunological homeostasis (1, 2). The transcription factor Foxp3 has been recognized as the leading signature gene and marker of Tregs (3), and reduced and unstable Foxp3 expression affects Treg differentiation and function (4). The inflammatory milieu in the tissue microenvironment and pharmacological molecules that induce chronic endoplasmic reticulum stress (ERS) could damage Treg stability and convert these Tregs to T effector cell–like (Teff cell–like) phenotypes that promote the activation and expansion of autoreactive T cells and other innate immune cells (5–8). However, the mechanisms underlying how the ERS response regulates Foxp3 stability and Treg function remain elusive.

The ER functions as the hub for the folding and quality control (QC) of secretory proteins, where misfolded proteins are primarily recognized by ER-associated degradation (ERAD) for proteasomal degradation (9, 10). Stress conditions in which mammalian cells are activated and increasingly require new protein synthesis can cause an accumulation of unfolded and misfolded proteins, identified by the ERS sensors inositol-requiring enzyme 1α (IRE1α), protein kinase R–like ER kinase (PERK), and activation of transcription factor 6 (ATF6) (11). IRE1α itself has been reported as an endogenous substrate for the ERAD complex that involves the E3 ubiquitin ligase HRD1 and the unfolded protein response (UPR) chaperone binding immunoglobulin protein (BIP) (12). However, the mechanism and function of ERAD-mediated degradation of IRE1α in Tregs require further investigation.

Several studies have revealed functions of the ERS response in T cells (13–17) or Tregs (8, 18). Unlike conventional CD4^+^ T cells, Tregs are mainly managed by mitochondrial oxidative phosphorylation (OXPHOS) for their bioenergetic needs (19–22). Dysregulated ERS responses and defects in mitochondrial OXPHOS are the main sources of cellular ROS (23, 24), which are subjected to clearance by nuclear factor erythroid 2-related factor 2 (NRF2), the best-described antioxidant transcription factor (25, 26). Adequate mitochondrial OXPHOS and ROS levels are critical for the regulation of Treg function (27–30), and it has been suggested that an ERS-induced UPR is highly dependent on the redox signaling response (31). However, the crosstalk among the ERS response, mitochondrial OXPHOS, and antioxidant response in Tregs remains elusive.

Yeast p24 protein can prevent misfolded proteins from entering vesicles secreted from the ER (32, 33). The human homolog of the p24 trafficking protein 4 (TMED4), namely ERS response protein 25 (ERS25), is a potential ERS response regulator. The ER Golgi transport inhibitor brefeldin A induced TMED4 expression, indicating that TMED4 may act as a vesicular transport protein between the ER and Golgi apparatus (34).

In our study, we found that, compared with their littermate controls, Treg-specific *Tmed4*-KO mice (*Tmed4^ΔTreg^*) had more Tregs, yet with lower Foxp3 expression and impaired suppressive activity. *Tmed4^ΔTreg^* mice exhibited exacerbated symptoms in experimental autoimmune encephalomyelitis (EAE), chronic colitis models and boosted antitumor immunity. Loss of TMED4 in Tregs led to defects in homeostasis of the ERS response and mitochondrial integrity, which caused accumulated cellular ROS and subsequently impaired Foxp3 stability and the suppressive function of Tregs, which could be restored by ROS scavenger, NRF2 inducer, or expression of IRE1α. Collectively, we found that TMED4 regulated Treg stability and suppressive function by sustaining IRE1α-dependent regulation of the cellular ROS and NRF2-related antioxidant response and that TMED4 suppressed IRE1α proteosome degradation via the ERAD system including BIP.

## Results

### Treg-specific Tmed4–KO (Tmed4^ΔTreg^) mice exhibit T cell hyperactivation and impaired Foxp3 stability in Tregs.

Within the tumor microenvironment, ERS plays a complex role in the balance between the antitumor activities of immune cells and tumor cell adaptation and escape, in which innate immunosuppressive profiles of Tregs can be reprogrammed to disturb their antitumor effect (35). We speculate that suppressive activity of tumor infiltrating Tregs correlates with their intrinsic ERS responses. To identify the potential influence of TMED4 on the regulatory function of Tregs within the tumor microenvironment, we conducted correlation analyses of the gene expression profiles obtained from various cancer datasets including colon adenocarcinoma (COAD), rectal adenocarcinoma (READ), lung adenocarcinoma (LUAD), liver hepatocellular carcinoma (LIHC), and gastric cancers (stomach adenocarcinoma [STAD]) sourced from The Cancer Genome Atlas (TCGA). We found a strong positive correlation between the expression profiles of ERS response–associated genes and *TMED4* (Supplemental Figure 1A; supplemental material available online with this article; https://doi.org/10.1172/JCI179874DS1), suggesting that TMED4 may regulate ERS response within the tumor microenvironment. Additionally, we observed a significant positive correlation between the expression profiles of Treg signature genes and *TMED4* (Supplemental Figure 1B). To verify these findings, we sorted CD4^+^CD25^hi^ Tregs from human colorectal, gastric and renal cancer samples for the correlation analyses of mRNA expression. Indeed, we observed that the *TMED4* mRNA expression positively correlated with that of *HSPA5* and *DDIT3*, which reflect activation of the unfolded protein response (UPR) (Supplemental Figure 1C). We further identified positive correlations between the expression levels of *TMED4*, *DDIT3*, or *HSPA5* (not significant but a correlation tendency) with *FOXP3* (Supplemental Figure 1D). The above evidence suggests that TMED4 positively correlates with ERS responses and FOXP3 expression in tumor-infiltrating Tregs. This prompted us to further investigate the involvement of TMED4 in regulating the suppressive functions of Tregs and how this may affect antitumor activity through ERS responses.

**Figure 1:**
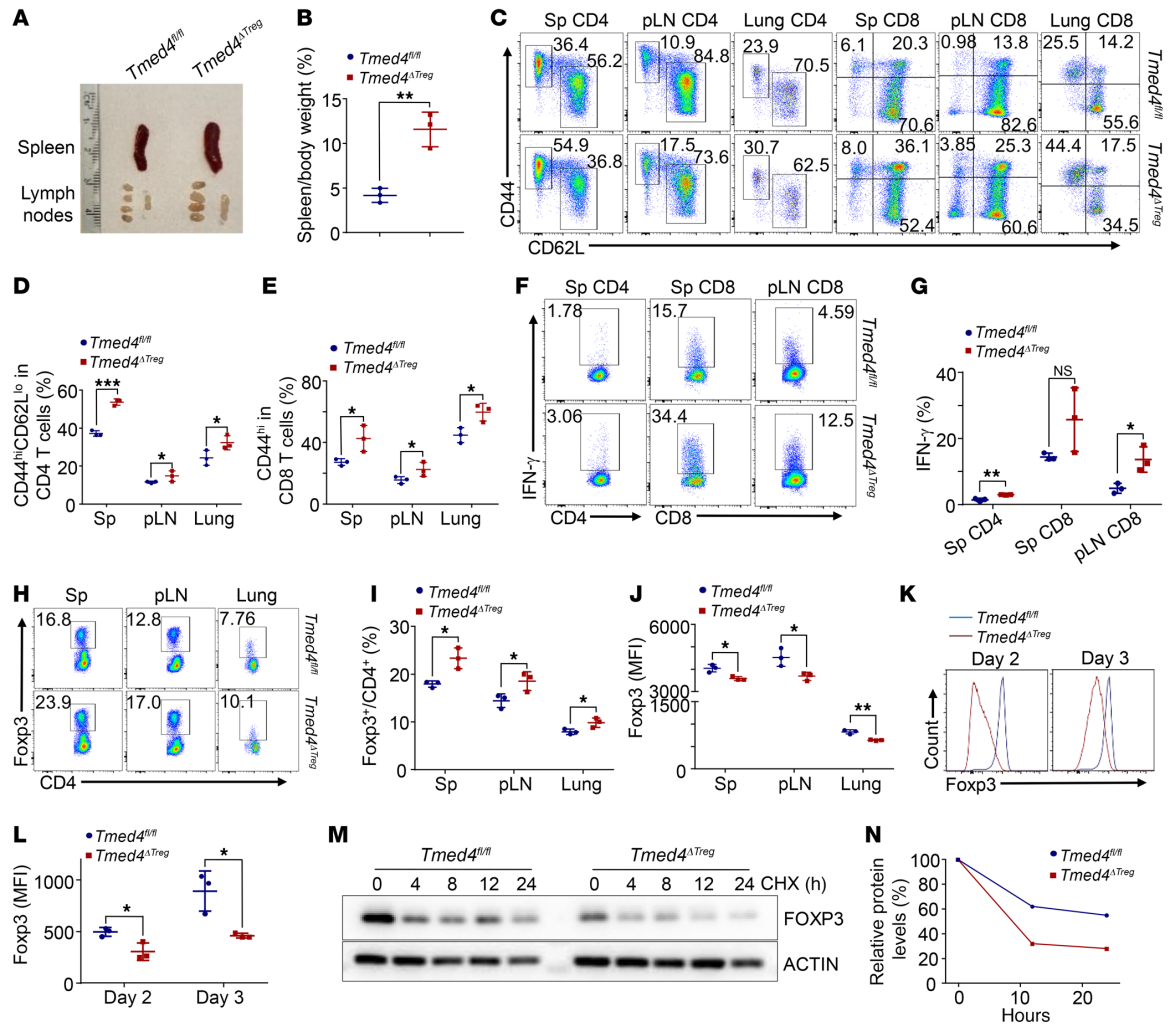
Treg-specific *Tmed4*-KO (*Tmed4^ΔTreg^*) mice exhibit T cell hyperactivation, and impaired Foxp3 stability in Tregs. (**A**) Images of spleens and pLNs from 12-week-old sex-matched littermate *Tmed4^fl/fl^* (WT) mice and *Tmed4^ΔTreg^* (cKO) mice. (**B**) Spleen/body weight ratios of *Tmed4^fl/fl^* and *Tmed4^ΔTreg^* mice (*n* = 3). (**C**–**E**) FCM plots (**C**) and analysis (**D** and **E**) of effector CD4^+^ and CD8^+^ T cells isolated from spleens, pLNs, and lungs from *Tmed4^fl/fl^* and *Tmed4^ΔTreg^* mice (*n* = 3). (**F** and **G**) FCM plots (**F**) and analysis (**G**) of IFN-γ–producing CD4^+^ and CD8^+^ T cells from spleens and pLNs from *Tmed4^fl/fl^* and *Tmed4^ΔTreg^* mice (*n* = 3). (**H** and **I**) FCM plots (**H**) and analysis (**I**) of Treg frequencies in CD4^+^ T cells from spleens, pLNs, and lungs from *Tmed4^fl/fl^* and *Tmed4^ΔTreg^* mice (*n* = 3). (**J**) FCM analysis of Foxp3 MFI in Tregs from spleens, pLNs, and lungs from *Tmed4^fl/fl^* and *Tmed4^ΔTreg^* mice (*n* = 3). (**K** and **L**) FCM levels (**K**) and analysis (**L**) of Foxp3 MFI in iTregs (CD4^+^CD25^+^Foxp3^+^) generated in vitro 2 or 3 days after the naive T cells were purified and cultured with differentiation inducers (*n* = 3). (**M** and **N**) Western blotting (**M**) and quantitative analysis (**N**) of FOXP3 decay in WT and *Tmed4*-deficient Tregs purified from spleens and pLNs treated with 1 μg/mL CHX for the indicated durations. Data are presented as the mean ± SEM of biologically independent samples and represent at least 3 independent experiments, each involving 3 mice per group. *P < 0.05, **P < 0.01, and ***P < 0.001, by 2-tailed Student’s *t* test. Sp, spleen.

We first generated mice with Treg-specific KO of *Tmed4* (*Tmed4^ΔTreg^*, also designated as cKO) by crossing *Foxp3-Cre* mice with *Tmed4^fl/fl^* mice, whose Tregs were confirmed to have efficient deletion of TMED4 in both protein and mRNA (Supplemental Figure 2, A and B). *Tmed4^ΔTreg^* mice started to show spontaneous inflammation and a propensity for autoimmunity as early as 12 weeks of age. Compared with their *Tmed4^fl/fl^* littermate controls, *Tmed4^ΔTreg^* mice exhibited splenomegaly, lymphadenopathy, lower body weight, and increased lymphocytic infiltration in nonlymphoid organs, particularly in the lungs (Figure 1, A and B, and Supplemental Figure 2C). Under resting conditions, spleens, peripheral lymph nodes (pLNs), and lungs of *Tmed4^ΔTreg^* mice contained higher percentages of CD4^+^CD44^hi^CD62^lo^ and CD8^+^CD44^hi^ Teff cells than did their littermate WT control mice (Figure 1, C–E). In addition, *Tmed4^ΔTreg^* mice had markedly higher frequencies of IFN-γ–producing CD4^+^ and CD8^+^ T cells in the spleen and pLNs than did *Tmed4^fl/fl^* mice (Figure 1, F and G). Furthermore, *Tmed4^ΔTreg^* mice had a relatively higher percentage of neutrophils (CD11b^+^Ly6G^+^) in the spleen than did *Tmed4^fl/fl^* mice (Supplemental Figure 2D). Thus, TMED4 deficiency in Tregs resulted in T cell hyperactivation and an inflammatory phenotype in mice.

We further assessed how TMED4 regulates the functions of Tregs. Compared with their littermate controls, *Tmed4^ΔTreg^* mice contained higher percentages of Tregs in the spleen, pLNs, and lungs (Figure 1, H and I). An in vitro assay showed that splenic *Tmed4*-deficient Tregs were hyperproliferative despite having Ki67^+^ levels similar to those of Tregs from WT controls (Supplemental Figure 2, E and F). The apoptosis ratios of Tregs from the spleens of mice of both genotypes were comparable when assessed using annexin V antibodies (Supplemental Figure 2, G and H), suggesting that the increased frequency of Tregs in *Tmed4^ΔTreg^* mice may not have been due to impairment in their ability to proliferate and survive, but rather to an impairment of the immunosuppressive function of Tregs that triggered a compensatory increase in Treg numbers in vivo. To further understand the intrinsic alterations in the stability and function of *Tmed4*-deficient Tregs, we found that the MFI of Foxp3 was reduced in KO Tregs from spleens, pLNs, and lungs (Figure 1J). In addition, induction of purified naive CD4^+^ T cells using Treg differentiation supplements for 2 and 3 days revealed that Foxp3 expression was lower in KO induced Tregs (iTregs) compared with expression in WT cells (Figure 1, K and L). Next, we measured FOXP3 protein decay by cycloheximide (CHX) treatment in mouse primary Tregs and found that the protein stability of FOXP3 in *Tmed4*-deficient Tregs was markedly reduced (Figure 1, M and N). In conclusion, TMED4 played a pivotal role in the maintenance of homeostasis and suppressive function of Tregs.

### Tmed4 deficiency alters the signature profiles of Tregs in a cell-intrinsic manner.

**Figure 2:**
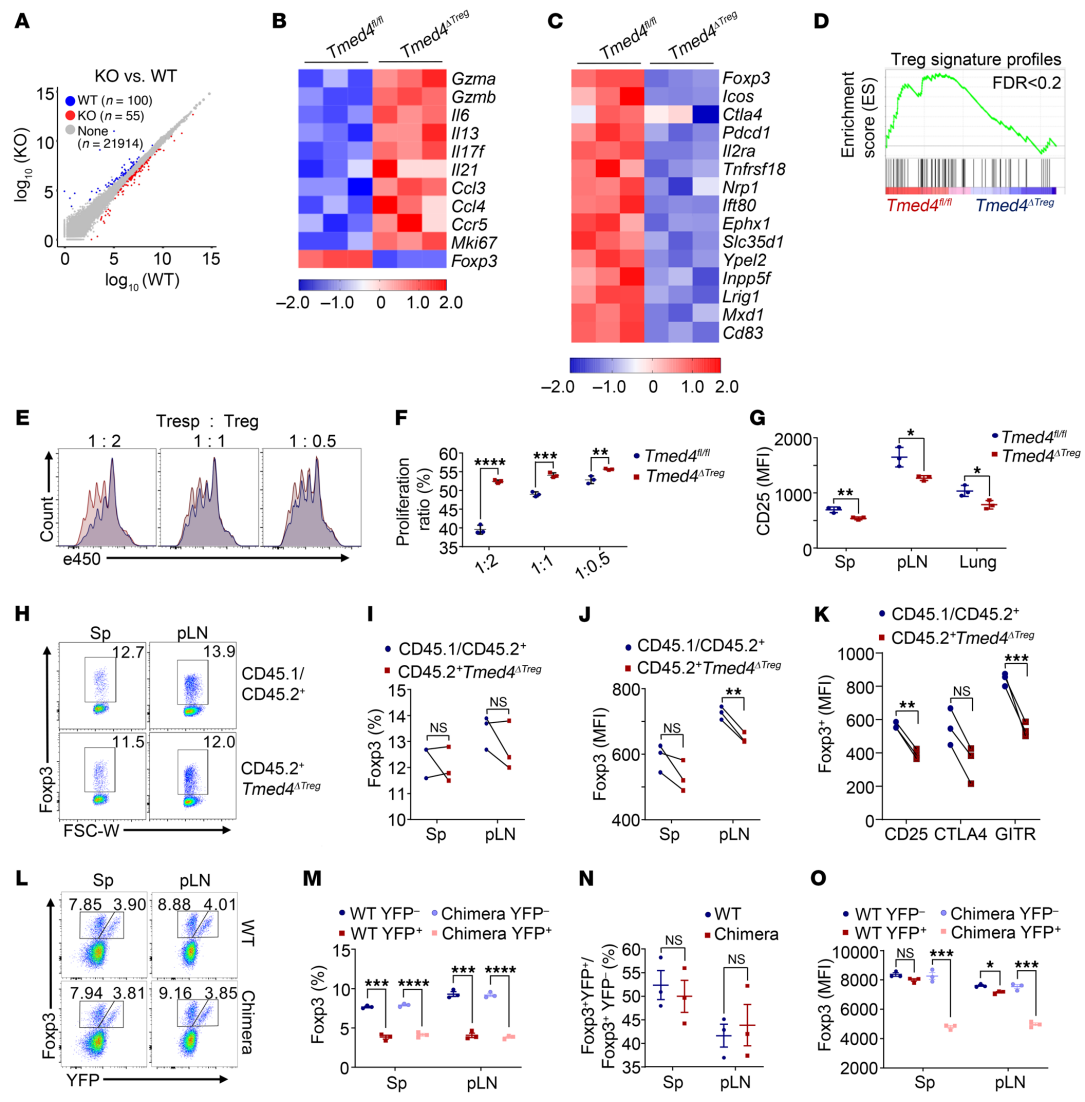
TMED4 deficiency alters the signature profiles of Tregs in a cell-intrinsic manner. (**A**) Scatterplot showing global gene expression profiles of Tregs from *Tmed4^fl/fl^* and *Tmed4^ΔTreg^* mice stimulated with α-CD3/α-CD28 antibodies. Transcripts with a |log_2_(fold change)| >0.5 and *P* < 0.05 in *Tmed4*-deficient Tregs are shown in blue or red. (**B** and **C**) Heatmaps of gene clusters of DEGs of effector-like genes (**B**) and Treg signature profile genes (**C**) and between WT and *Tmed4*-deficient Tregs. Red and blue represent relatively higher and lower levels of expression of the indicated genes, respectively. The colors indicate the value of the log_2_ fold change (*n* = 3). (**D**) GSEA plots showing the enrichment of the “Treg signature profiles” (FDR <0.2) gene set. (**E** and **F**) FCM levels (**E**) and statistical analysis (**F**) of the in vitro suppressive assay of purified Tregs from spleens from *Tmed4^fl/fl^* and *Tmed4^ΔTreg^* mice, as assessed by the proliferation of activated CD4^+^ T cells in the presence of various ratios (responder T cells/Tregs [Tresp/Treg] = 1:2, 1:1, and 1:0.5) of Tregs (*n* = 3, detected on day 3). (**G**) FCM analysis of MFI of CD25 in Tregs from spleens, pLNs, and lungs of *Tmed4^fl/fl^* and *Tmed4^ΔTreg^* mice (*n* = 3). (**H** and **I**) FCM plots (**H**) and analysis (**I**) of Foxp3^+^ Treg frequencies in BM chimeric mice (*n* = 3). FSC-W, forward scatter width. (**J** and **K**) FCM analysis of Foxp3 (**J**), CD25, CTLA4, and GITR (**K**) MFI in spleens and pLNs from BM chimeric mice (*n* = 3). (**L** and **M**) FCM plots (**L**) and Foxp3 frequencies (**M**) of both YFP^+^ and YFP^–^ Tregs in spleens and pLNs from female WT and chimeric mice (*n* = 3). (**N**) Ratio of YFP^+^ Tregs to YFP^–^ Tregs in spleens and pLNs from female WT and chimeric mice (*n* = 3). (**O**) FCM analysis of Foxp3 MFI between YFP^+^ and YFP^–^ Tregs from female WT and chimeric mice (*n* = 3). Data are presented as the mean ± SEM of biologically independent samples and represent at least 3 independent experiments, each involving 3 mice per group. *P < 0.05, **P < 0.01, ***P < 0.001, and ****P < 0.0001, by 2-tailed Student’s *t* test.

To systematically investigate the effect of *Tmed4* deficiency on Treg homeostasis and function, we performed RNA-Seq analysis of in vitro–activated Tregs, which showed differentially expressed genes (DEGs) (55 upregulated genes and 100 downregulated genes) in *Tmed4*-deficient Tregs (Figure 2A). Of particular note, we detected markedlyenhanced expression of genes such as *Gzma*, *Gzmb*, *Il6*, *Il17f*, *Il13*, and *Ccl3*, reflecting Teff cell function and differentiation in KO Tregs (Figure 2B). Meanwhile, the signature genes reflecting Treg stability and function were markedly downregulated in the *Tmed4*-deficient group (Figure 2, C and D). Since *Tmed4* deletion impaired the stability of Foxp3, we subsequently confirmed that the absence of *Tmed4* also downregulated the immunosuppressive function of Tregs against T cell proliferation by in vitro suppression assays (Figure 2, E and F). We also found that the MFI of CD25, a key molecule that characterizes Tregs, was reduced in KO Tregs from spleens, pLNs, and lungs (Figure 2G)

To rule out the possible influence of extrinsic inflammation on Tregs in vivo, we generated bone marrow (BM) chimeric mice, in which recipient CD45.1^+^ mice were subjected to x-ray irradiation to eliminate hematopoietic cells, followed by reconstitution of these cells with a mixture of BM cells from CD45.1/CD45.2^+^ *Tmed4^fl/fl^* mice and CD45.2^+^ *Tmed4^ΔTreg^* mice. In these mice, the Treg percentages were comparable (Figure 2, H and I), and expression levels of representative surface markers of Tregs, including Foxp3, CD25, CTLA4, and GITR, in *Tmed4*-deficient Tregs were all reduced compared with levels in their WT counterparts (Figure 2, J and K). In particular, *Tmed4*-deficient Tregs expressed higher levels of programmed cell death 1 (PD-1), which is generally considered to be negatively correlated with suppressive function (36). Although the upregulation of CD69 showed higher activation of *Tmed4*-deficient Tregs, OX40 and ICOS were downregulated (Supplemental Figure 3, A and B). To further determine whether TMED4 regulates Treg homeostasis in a cell-intrinsic manner, we used *Tmed4^fl/fl Foxp3Cre/+^* female mice (designated as chimeric mice), in which approximately half of the Tregs were TMED4 sufficient (without *Foxp3*-driven YFP-Cre fusion protein expression, designated as YFP^–^ Tregs), while the other half of the Tregs were *Tmed4* deficient (with Foxp3-driven YFP-Cre fusion protein expression, designated as YFP^+^ Tregs) as a result of random X chromosome inactivation (37). In both *Tmed4^+/+ Foxp3Cre/+^* mice (both YFP^–^ and YFP^+^ Tregs were *Tmed4* sufficient) and chimeric mice, although the proportion of YFP^+^ Tregs was lower than that of YFP^–^ Tregs (Figure 2, L and M), the relative ratios of YFP^+^ to YFP^–^ Tregs were similar between the 2 groups (Figure 2N), suggesting that loss of *Tmed4* did not affect the proliferative properties of Tregs in the chimeric mice. However, the expression of Foxp3 as well as CD25, CTLA4, and GITR was significantly downregulated only in the chimera YFP^+^ Tregs (Figure 2O and Supplemental Figure 3C). Taken together, our observations suggested that *Tmed4*-deficient Tregs had an effector phenotype and reduced signature profiles that were cell intrinsic.

### Loss of Tmed4 in Tregs leads to a more exacerbated inflammatory phenotype and boosts antitumor immunity in mice.

**Figure 3:**
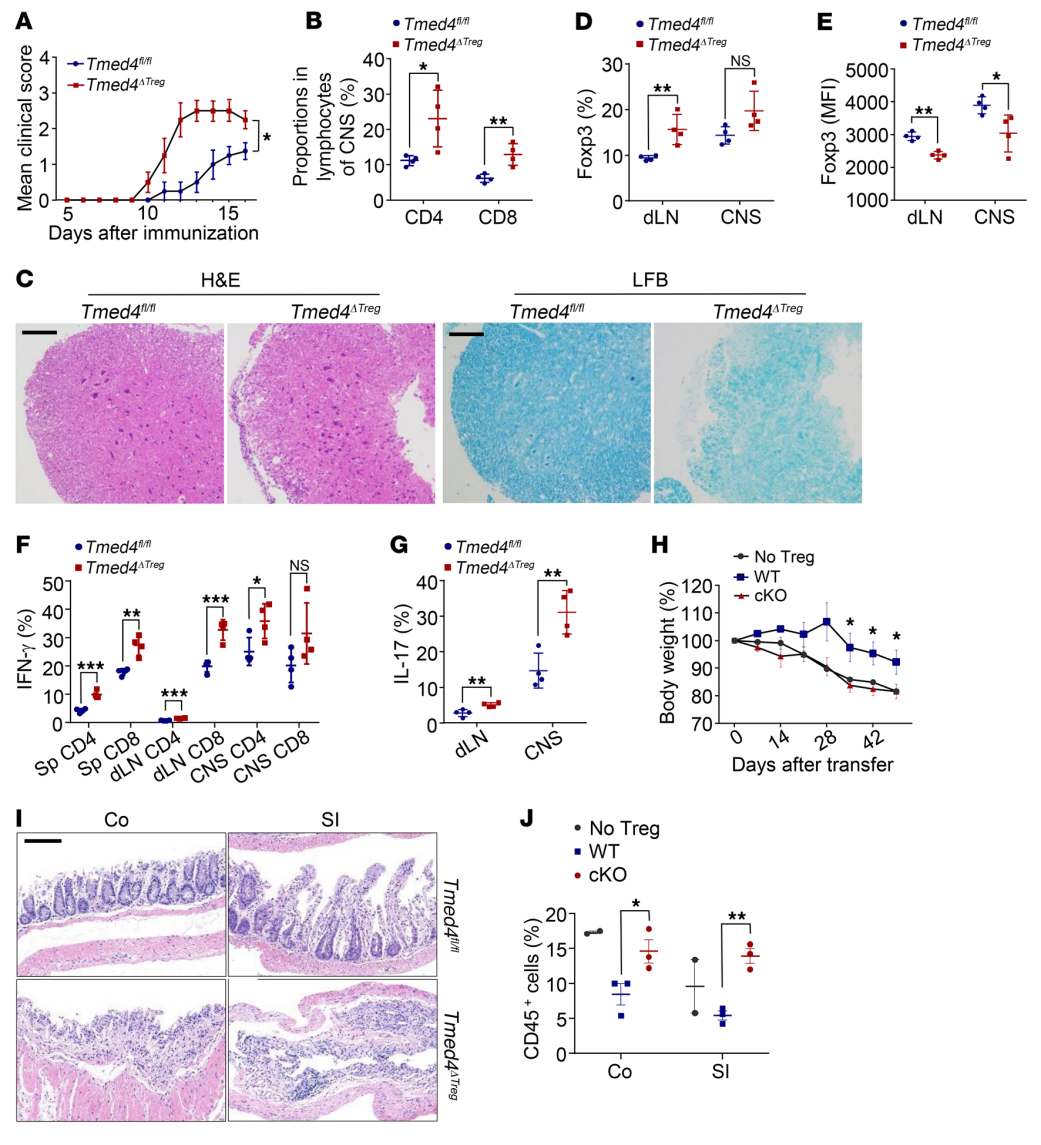
Loss of *Tmed4* in Tregs leads to a more exacerbated inflammatory phenotype in mice. (**A**) Mean clinical score for diseased *Tmed4^fl/fl^* and *Tmed4^ΔTreg^* mice (*n* = 4). (**B**) FCM analysis of CD4^+^ and CD8^+^ T cell proportions isolated from the CNS of diseased *Tmed4^fl/fl^* and *Tmed4^ΔTreg^* mice 16 days after disease induction (*n* = 4). (**C**) H&E and LFB stainings of spinal cord sections from diseased *Tmed4^fl/fl^* and *Tmed4^ΔTreg^* mice (16 days after immunization). Scale bars: 100 μm. Samples were selected from 1 *Tmed4^fl/fl^* mouse (clinical score: 1) and 1 *Tmed4^ΔTreg^* mouse (clinical score: 3). (**D** and **E**) FCM analysis of Treg frequencies (**D**) and Foxp3 MFI (**E**) in dLNs and CNS (*n* = 4). (**F** and **G**) FCM analysis of (**F**) IFN-γ–producing CD4^+^ and CD8^+^ T cells from spleens, dLNs, and CNS and (**G**) IL-17–producing CD4^+^ T cells from dLNs and CNS from diseased *Tmed4^fl/fl^* and *Tmed4^ΔTreg^* mice (*n* = 4). (**H**) Curve for the percentage of body weight loss. *Rag1*^–/–^ mice were injected with CD4^+^CD45RB^hi^CD25^lo^ naive T cells alone or in combination with Tregs isolated from *Tmed4^fl/fl^* and *Tmed4^ΔTreg^* mice. The body weight is presented relative to the initial weight in each case (*n* = 3 for mice coinjected with naive T mixed *Tmed4^fl/fl^* or *Tmed4^ΔTreg^* Tregs, respectively; *n* = 2 for mice injected with naive T cells alone). (**I**) H&E staining of colon (Co) and small intestine (SI) after adoptive transfer. Scale bar: 100 μm. (**J**) Percentages of CD45^+^ cells infiltrating into the colon and small intestine (*n* = 3 for mice coinjected with naive T mixed WT or *Tmed4*-deficient Tregs, respectively; *n* = 2 for mice injected with naive T cells alone). Data are presented as the mean ± SEM of biologically independent samples and represent 3 independent experiments, each involving 2–4 mice per group. *P < 0.05, **P < 0.01, and ***P < 0.001, by 2-way ANOVA (**A**), 1-way ANOVA with Tukey’s multiple-comparison test (**H**), and 2-tailed Student’s *t* test for other analysis.

To investigate the in vivo pathophysiological consequences of *Tmed4* ablation in Tregs, we used an EAE mouse model. In this model, mice exhibit clinical EAE scores upon myelin oligodendrocyte glycoprotein (MOG) peptide immunization. In the parallel treatment groups, *Tmed4^ΔTreg^* mice developed more severe disease symptoms than did the littermate *Tmed4^fl/fl^* control mice 11 days after immunization (Figure 3A and Supplemental Figure 4A) and had higher CNS infiltration of CD4^+^ and CD8^+^ T cells (Figure 3B). Through H&E staining and Luxol fast blue (LFB) histological staining analysis, we confirmed that *Tmed4^ΔTreg^* mice had more CNS-infiltrating immune cells and more severe demyelination than did *Tmed4^fl/fl^* group (Figure 3C). Consistent with the resting conditions results, the draining lymph nodes (dLNs) and CNS of *Tmed4^ΔTreg^* mice contained higher frequencies of Tregs, but lower Foxp3 expression, than that was observed in their *Tmed4^fl/fl^* controls (Figure 3, D and E, and Supplemental Figure 4B). Consequently, the percentages of IFN-γ–secreting CD4^+^ or CD8^+^ T cells and IL-17–secreting CD4^+^ T cells were also higher in both the dLNs and CNS of *Tmed4^ΔTreg^* mice (Figure 3, F and G, and Supplemental Figure 4, C and D).

We further used a T cell transfer colitis model, in which naive T cells administered to lymphopenic *Rag1^–/–^* mice resulted in severe colitis, while cotransfer of naive T cells together with Tregs attenuated the severity of colitis (38). *Rag1^–/–^* mice transferred with naive T cells and *Tmed4*-deficient Tregs showed progressive loss of body weight, as was observed with cotransfer of T cells in the naive and WT Treg groups (Figure 3H), with the latter group also exhibiting less intestinal damage, as confirmed by H&E histological analysis (Figure 3I), and significantly less inflammatory cell infiltration in both the colon and small intestine (Figure 3J). To rule out a role for mutant Tregs in the development of the disease, we injected either WT or KO Tregs into *Rag1^–/–^* mice and found that neither group of Tregs induced significant colitis like CD4^+^ T cell injection did (Supplemental Figure 4E).

**Figure 4:**
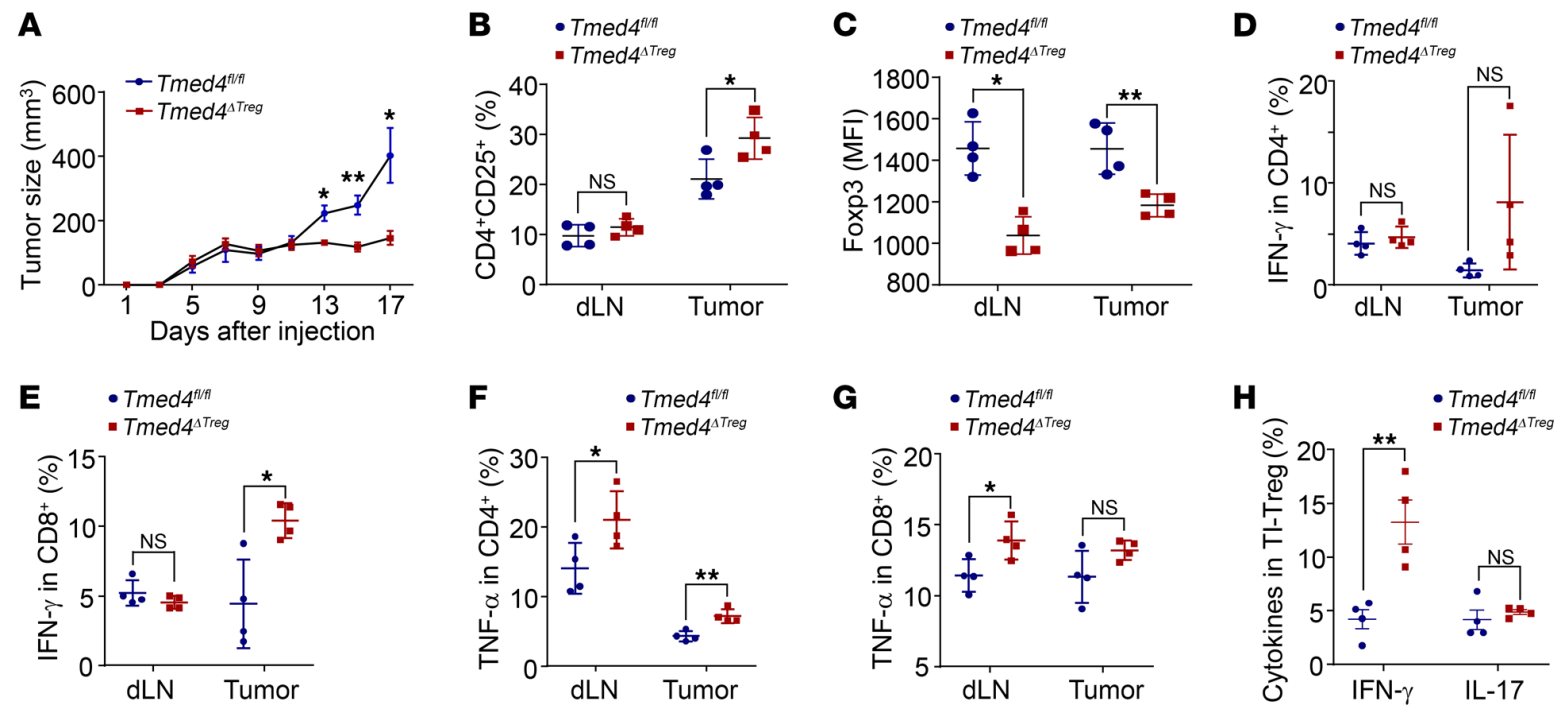
Loss of *Tmed4* in Tregs boosts antitumor immunity in mice. (**A**) Growth curves for tumors derived from *Tmed4^fl/fl^* and *Tmed4^ΔTreg^* mice that were s.c. inoculated with MC38 cells (*n* = 4). (**B** and **C**) FCM analysis of CD4^+^CD25^+^ frequencies (**B**) and Foxp3 MFI (**C**) of dLNs and tumor-infiltrating CD4^+^Foxp3^+^ Tregs from tumor-bearing *Tmed4^fl/fl^* and *Tmed4^ΔTreg^* mice (*n* = 4). (**D**–**G**) FCM analysis of the percentages of IFN-γ (**D** and **E**) and TNF-α (**F** and **G**) secretion in tumor-infiltrating CD4^+^ and CD8^+^ T cells from dLNs and tumors from *Tmed4^fl/fl^* and *Tmed4^ΔTreg^* mice (*n* = 4). (**H**) Percentages of IFN-γ– and IL-17–producing, tumor-infiltrating Tregs (TI-Treg) from tumor-bearing *Tmed4^fl/fl^* and *Tmed4^ΔTreg^* mice (*n* = 4). Data are presented as the mean ± SEM of biologically independent samples and represent 3 independent experiments, each involving 4 mice per group. *P < 0.05 and **P < 0.01, by 2-tailed Student’s *t* test

Since the manifestation of TMED4 is significantly associated with Foxp3 in human tumors, we proceeded to investigate how TMED4 regulates Treg-mediated antitumor immunity. The colorectal cancer MC38 cells inoculated into *Tmed4^ΔTreg^* mice had significantly slower growth kinetics and resulted in smaller tumor sizes compared with their littermate controls (Figure 4A). Consistently, T cell activation was significantly higher in lymphoid organs, particularly in dLNs of *Tmed4^ΔTreg^* mice (Supplemental Figure 5, A–D). The proportion of tumor-infiltrating Tregs was higher in tumor-bearing *Tmed4^ΔTreg^* mice (Figure 4B and Supplemental Figure 5E), whose Tregs derived from dLNs, and the tumors had significantly lower Foxp3 expression levels (Figure 4C). Intracellular staining (ICS) revealed significantly higher IFN-γ levels or a greater tendency for production of IFN-γ (Figure 4, D and E, and Supplemental Figure 5F) and TNF-α (Figure 4, F and G, and Supplemental Figure 5G) by T cells, especially in tumor-infiltrating T cells derived from *Tmed4^ΔTreg^* mice. The results also showed that tumor-infiltrating Tregs derived from *Tmed4^ΔTreg^* mice secreted more IFN-γ, but no differences in IL-17 levels were observed (Figure 4H and Supplemental Figure 5H). Collectively, loss of *Tmed4* in Tregs led to a more exacerbated inflammatory phenotype and boosted antitumor immunity in mice.

### Tmed4 deficiency leads to an impaired ERS response, mitochondria integrity, and ROS accumulation in Tregs.

**Figure 5:**
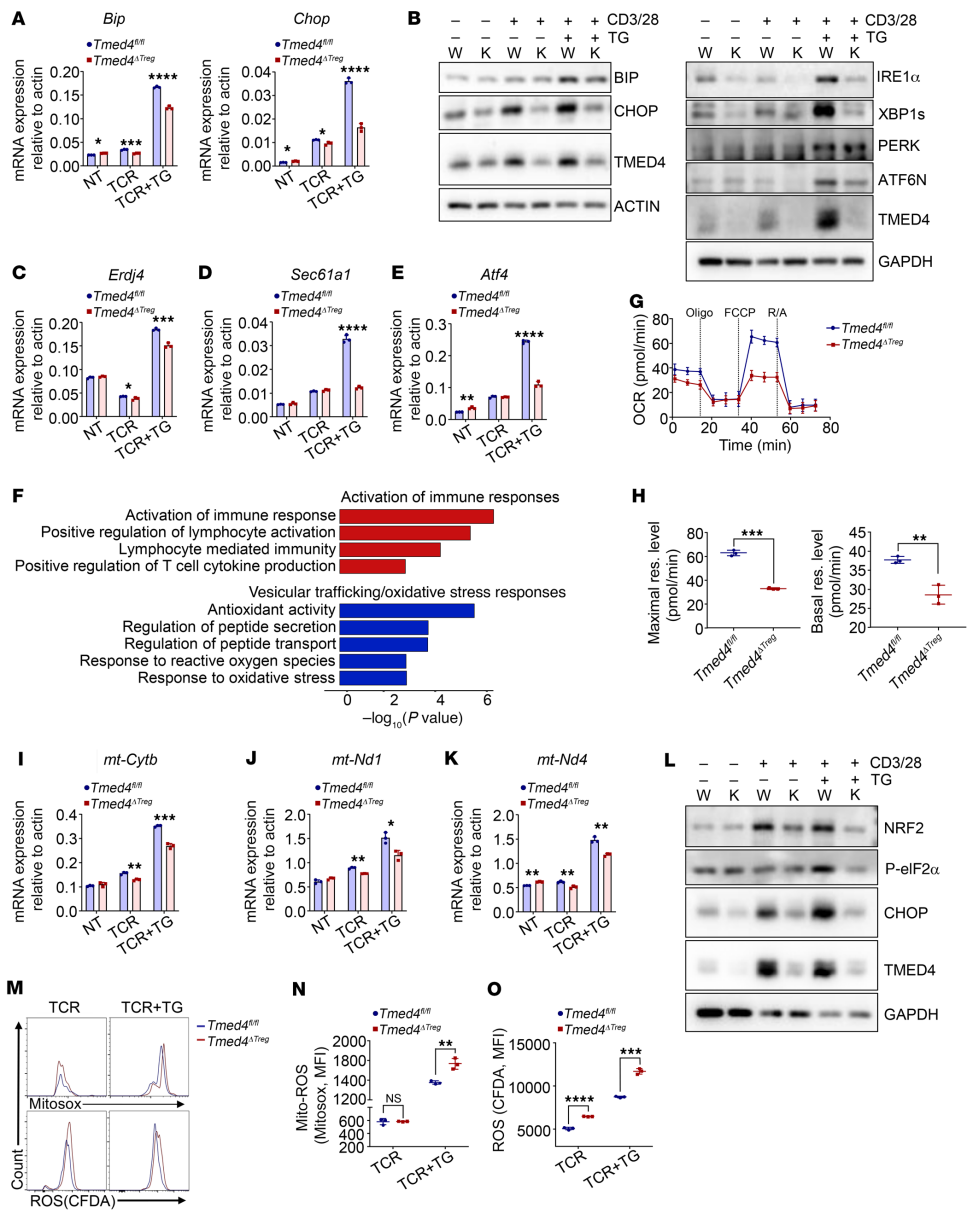
*Tmed4* deficiency leads to an impaired ERS response, mitochondrial integrity, and ROS accumulation in Tregs. (**A**) Relative mRNA expression of *Bip* and *Chop* in WT and *Tmed4*-deficient Tregs under resting conditions or with α-CD3/α-CD28 antibodies (TCR), alone or together with 1 μM TG to induce ERS, for 16–20 hours (*n* = 3). (**B**) WB analysis of ERS-related regulator proteins and their downstream molecules in WT and *Tmed4*-deficient Tregs treated with DMSO, α-CD3/α-CD28 antibodies only or together with 1 μM TG for 16–20 hours. (**C**–**E**) Relative mRNA expression of *Erdj4* (**C**), *Sec61a1* (**D**), and *Atf4* (**E**) in WT and *Tmed4*-deficient Tregs under resting conditions or with α-CD3/α-CD28 antibodies (TCR), alone or together with 1 μM TG to induce ERS, for 16–20 hours (*n* = 3). (**F**) Enrichment analysis of KEGG and GO pathways for *Tmed4*-deficient Tregs compared with WT Tregs. Selected upregulated (red bars) and downregulated (blue bars) pathways are shown for *Tmed4*-deficient Tregs. (**G** and **H**) Curve (**G**) and quantitative analysis (**H**) of the OCRs for WT and *Tmed4*-deficient Tregs stimulated with α-CD3/α-CD28 for 16–20 hours (*n* = 3). (**I**–**K**) Relative mRNA expression of *mt-Cytb* (**I**), *mt-Nd1* (**J**), and *mt-Nd4* (**K**) in WT and *Tmed4*-deficient Tregs under resting conditions or with α-CD3/α-CD28 antibodies (TCR), or together with 1 μM TG stimulation for 16–20 hours (*n* = 3). (**L**) WB analysis of PERK pathway–related proteins in WT and *Tmed4*-deficient Tregs treated with α-CD3/α-CD28 antibodies, alone or together with 1 μM TG, for 16–20 hours. (**M**–**O**) Levels of mitochondrial ROS (Mito-ROS) (**M**, upper panel) and total cellular ROS (**M**, lower panel) stained with Mito-SOX and CFDA (H2DCFDA), respectively, and quantitative analysis of their MFI (**N** and **O**) in WT and *Tmed4*-deficient splenic Tregs stimulated for 16–20 hours with α-CD3/α-CD28 antibodies (TCR), alone or together with 1 μM TG (*n* = 3). Data are presented as the mean ± SEM of biologically independent samples and represent at least 3 independent experiments, each involving 3 mice per group. *P < 0.05, **P < 0.01, ***P < 0.001, and ****P < 0.0001, by 2-tailed Student’s *t* test. K, KO group; WT, WT group.

Given that TMED4 functions as an ERS response protein and imbalanced ERS response would cause Tregs instability and dysfunction (8, 34), we set out to study how TMED4 may regulate ERS responses in Tregs. We induced ERS in vitro using thapsigargin (TG), an inhibitor of microsomal Ca^2+^-ATPase. Upon T cell receptor (TCR) activation and ERS induction, the UPR activation hallmark genes *Bip* and *Chop* were significantly upregulated in the WT Tregs, yet the extent of induction was dramatically decreased in *Tmed4*-deficient Tregs (Figure 5A). Importantly, *Tmed4* deficiency in Tregs led to significant downregulation of IRE1α protein levels and XBP1 spliced form XBP1s, whereas ATF6N (cleaved N-terminus of ATF6, representing its activated form) and PERK were only slightly reduced or unchanged (Figure 5B). In keeping with the reduction of IRE1α protein levels, *Tmed4*-deficient Tregs also had lower levels of phosphorylated IRE1α and TRAF2 protein (Supplemental Figure 6A). In addition to the typical ERS markers, the transcription levels of the *Xbp1* downstream target genes *Erdj4* and *Sec61a1* and the PERK downstream gene *Atf4* were all significantly downregulated (Figure 5, C–E).

ERS could affect many facets of mitochondrial functions, through which the IRE1α and PERK arms could affect mitochondrial bioenergetics and proteostasis regulation through mitochondrial QC (39–41). Recent studies have shown that ER dysfunction can raise the calcium and oxidative load in the ER and mitochondria, leading to mitochondrial dysfunction (23, 31). Kyoto Encyclopedia of Genes and Genomes (KEGG) and Gene Ontology (GO) analysis showed that genes associated with the activation of immune response pathways were upregulated, whereas genes associated with vesicle transport and antioxidant response pathways in *Tmed4*-deficient Tregs were downregulated (Figure 5F). Seahorse analysis showed decreased oxygen consumption rates (OCRs) in KO Tregs (Figure 5, G and H), indicating a deficit in the capacity for mitochondrial activity. In addition, we detected a higher degree of mitochondrial damage in KO Tregs, as shown by Mito-Tracker FCM staining (Supplemental Figure 6B). Interestingly, we found upregulation of extracellular acidification rate (ECAR) levels in *Tmed4*-deficient Tregs (Supplemental Figure 6, C and D), which also had higher levels of mTOR phosphorylation and downstream S6 phosphorylation, as shown by FCM analysis (Supplemental Figure 6, E and F). The above phenotypes suggested that *Tmed4*-deficient Tregs may compensate for OXPHOS defects due to mitochondrial damage by glycolysis, and cell proliferation was facilitated by upregulation of mTOR pathway activity at the cost of Foxp3 expression and suppressive function, which matched the findings of a previous report (42).

Cellular imbalance of redox homeostasis caused by dysregulation of the ERS response and mitochondrial integrity leads to excessive accumulation of intracellular ROS, the well-recognized key factor affecting the biological processes of Tregs, and can further regulate the immunosuppressive functions of Tregs (27, 28). The production of ROS by mitochondria is modulated by complex I and complex III, which contribute to both oxidative respiration and the clearance of ROS generated from the mitochondrial electron transport chain (43). Analysis of RNA-Seq data revealed that expression levels of different components of mitochondrial complex genes were decreased in *Tmed4*-deficient Tregs (Supplemental Figure 6G). We also validated the defect in expression of several pivotal genes of mitochondrial complexes I and III, including *mt-Cytb*, *mt-Nd1*, and *mt-Nd4* in *Tmed4*-deficient Tregs (Figure 5, I–K). Activation of the antioxidant response, which is responsible for scavenging ROS, is dominantly facilitated by NRF2 (24, 44), known as a PERK substrate (45), along with factors associated with the antioxidant response. Correspondingly, we found a significant positive correlation between upregulation of TMED4 and activation of the antioxidant response in the tumor microenvironment, as obtained from TCGA datasets (Supplemental Figure 6H). PERK downstream kinase activity indicated by eIF2α phosphorylation and NRF2 protein levels were also markedly reduced (Figure 5L). Concomitantly, the mRNA levels of NRF2 downstream genes *Gclm* and *Ho-1* were also drastically reduced in *Tmed4*-deficient Tregs (Supplemental Figure 6, I and J). Next, we set out to detect mitochondrial ROS and cellular total ROS in splenic Tregs through Mito-SOX and CFDA staining, respectively. The results showed higher expression level of mitochondrial ROS and total ROS upon TCR stimulation and a more dramatical increase when ERS was induced in *Tmed4*-deficient Tregs (Figure 5, M–O). Collectively, TMED4 maintains the ERS response, mitochondrial integrity, and ROS homeostasis in Tregs.

### The ROS scavenger or NRF2 inducer restores Foxp3 expression and the suppressive function of Tmed4-deficient Tregs.

**Figure 6:**
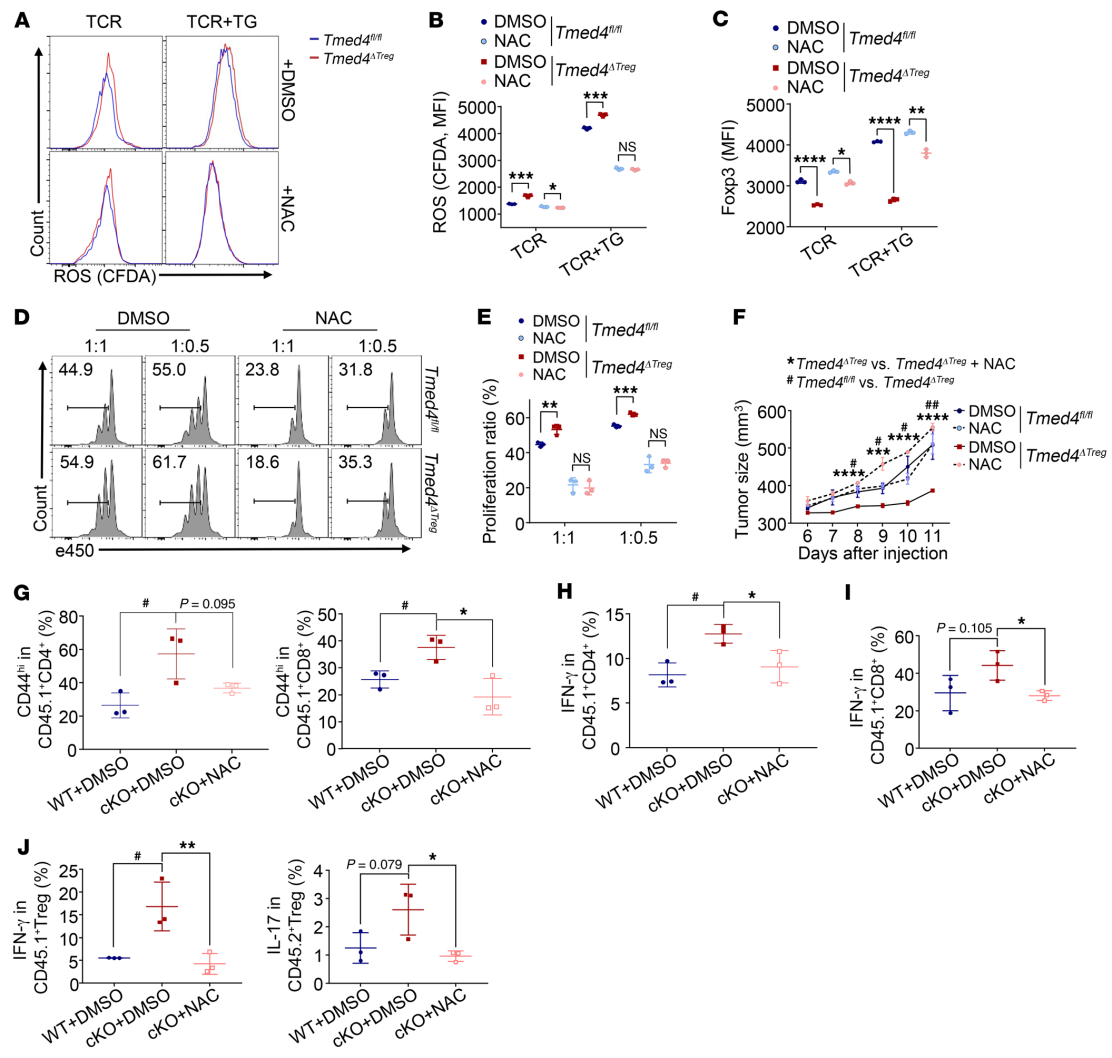
ROS scavenger or NRF2 inducer restores Foxp3 expression and the suppressive function of *Tmed4*-deficient Tregs. (**A**–**C**) Total cellular ROS levels (**A**) of splenic Tregs stimulated with TCR alone or together with 1 μM TG for 12 hours in the presence or absence of NAC (*N*-acetylcysteine) and quantitative analysis of the MFI of ROS (**B**) and Foxp3 (**C**) expression (*n* = 3). (**D** and **E**) FCM levels (**D**) and analysis (**E**) of the in vitro suppressive assay of purified Tregs from spleens and LNs from *Tmed4^fl/fl^* and *Tmed4^ΔTreg^* mice, as assessed by the proliferation of activated CD4^+^ T cells in the presence of the indicated ratios of Tregs pretreated with DMSO or NAC for 12 hours (*n* = 3, detected on day 3). (**F**) Tumor growth curves for CD45.1^+^ mice that were s.c. injected with MC38 cells, together with WT or *Tmed4*-deficient Tregs. Both types of Tregs were pretreated with α-CD3/α-CD28 antibodies for 12 hours in the presence or absence of NAC, and then i.v. injected into the mice on day 0 and day 7 (*n* = 4). (**G**) FCM analysis of the proportions of activated host CD45.1^+^CD4^+^ (**G**, left) and CD45.1^+^CD8^+^ (**G**, right) T cells from WT and KO mouse groups in the presence or absence of NAC, respectively (*n* = 3). (**H** and **I**) IFN-γ–producing host CD45.1^+^CD4^+^ (**H**) and CD45.1^+^CD8^+^ (**I**) T cells from WT and KO mouse groups in the presence or absence of NAC, respectively (*n* = 3). (**J**) IFN-γ– and IL-17–producing donor CD45.2^+^ Tregs from WT and KO mouse groups in the presence or absence of NAC, respectively (*n* = 3). Data are presented as the mean ± SEM of biologically independent samples. The tumor rescue model represents 2 independent experiments, and others represent 3 independent experiments, each involving 3–4 mice per group. *P < 0.05 or ^#^*P* < 0.05, **P < 0.01 or ^##^*P* < 0.01, ***P < 0.001, and ****P < 0.0001, by 1-way ANOVA with Tukey’s multiple-comparison test (**F**–**J**) and 2-tailed Student’s *t* test.

*Tmed4* deficiency led to ROS accumulation and impaired the antioxidant response in Tregs. It is therefore important to determine whether directly scavenging cellular ROS or restoring NRF2 activity could affect cellular phenotypes of *Tmed4*-deficient Tregs. We treated WT and *Tmed4*-deficient splenic Tregs with *N*-acetylcysteine (NAC), a common ROS scavenger, and found that the differences in ROS levels between these 2 genotypes of Tregs were significantly erased (Figure 6, A and B). TG-induced ERS promotes Treg-suppressive activity and Foxp3 expression to a certain extent (46). Foxp3 expression levels in *Tmed4*-deficient Tregs were achieved by NAC plus TG treatment, but not by NAC alone (Figure 6C). The same effect was achieved by the NRF2 inducer sulforaphane (Supplemental Figure 7, A–C), which could restore NRF2 levels in *Tmed4*-deficient Tregs similarly to those of WT Tregs (Supplemental Figure 7D). Interestingly, expression of Foxp3 in cells treated with higher doses of NAC (5 mM vs. 1 mM) was not restored but contrarily decreased in the presence of TCR stimulation alone, while Foxp3 expression was better restored when ERS was induced (Supplemental Figure 7, E and F). This finding indicates that appropriate ROS levels are essential for the maintenance of Foxp3 expression in Tregs. To further examine whether modulating ROS levels could erase the difference in the suppressive function of WT and *Tmed4*-deficient Tregs both in vitro and in vivo, we first performed an in vitro suppression assay and found that the suppressive function of both WT and *Tmed4*-deficient Tregs was rescued to the same degree (Figure 6, D and E). Meanwhile, we measured the levels of cellular ROS and found that NAC pretreatment could have a lasting suppressive effect on cellular ROS levels (Supplemental Figure 7, G and H). Additionally, we carried out in vivo rescue experiments in which WT or *Tmed4*-deficient CD45.2^+^ Tregs pretreated with DMSO and NAC, respectively, were inoculated into MC38 tumor cell–bearing CD45.1^+^ mice. Two consecutive inoculations of Tregs subjected to these different treatments were i.v. injected on days 0 and 7 (working model is shown in Supplemental Figure 8A). Tumor growth differences between the *Tmed4^fl/fl^* and *Tmed4^ΔTreg^* mice treated with DMSO were eliminated by NAC treatment (Figure 6F and Supplemental Figure 8B). Furthermore, we found that the activation of host CD4^+^ and CD8^+^ T cells in the NAC-pretreated group was effectively rescued, not only in terms of downregulation of the proportion of effector cells, but also secretion of IFN-γ (Figure 6, G–I, and Supplemental Figure 8, C–E). In addition, the percentages of both IFN-γ– and IL-17–secreting Tregs in the group that was inoculated with NAC were significantly decreased compared with those of nontreated *Tmed4*-deficient Tregs (Figure 6J and Supplemental Figure 8F). Meanwhile, cellular ROS levels showed that NAC could still provide sustained suppression of cellular ROS after a 7-day pretreatment (Supplemental Figure 8, G and H). The above results demonstrated that the cellular ROS scavenger or NRF2 activation restored the expression levels of Foxp3 and the suppressive activity of *Tmed4*-deficient Tregs both in vitro and in vivo.

### Tmed4 deficiency in Tregs leads to lower Foxp3 expression and ROS accumulation in an IRE1α/XBP1 axis–dependent manner.

**Figure 7:**
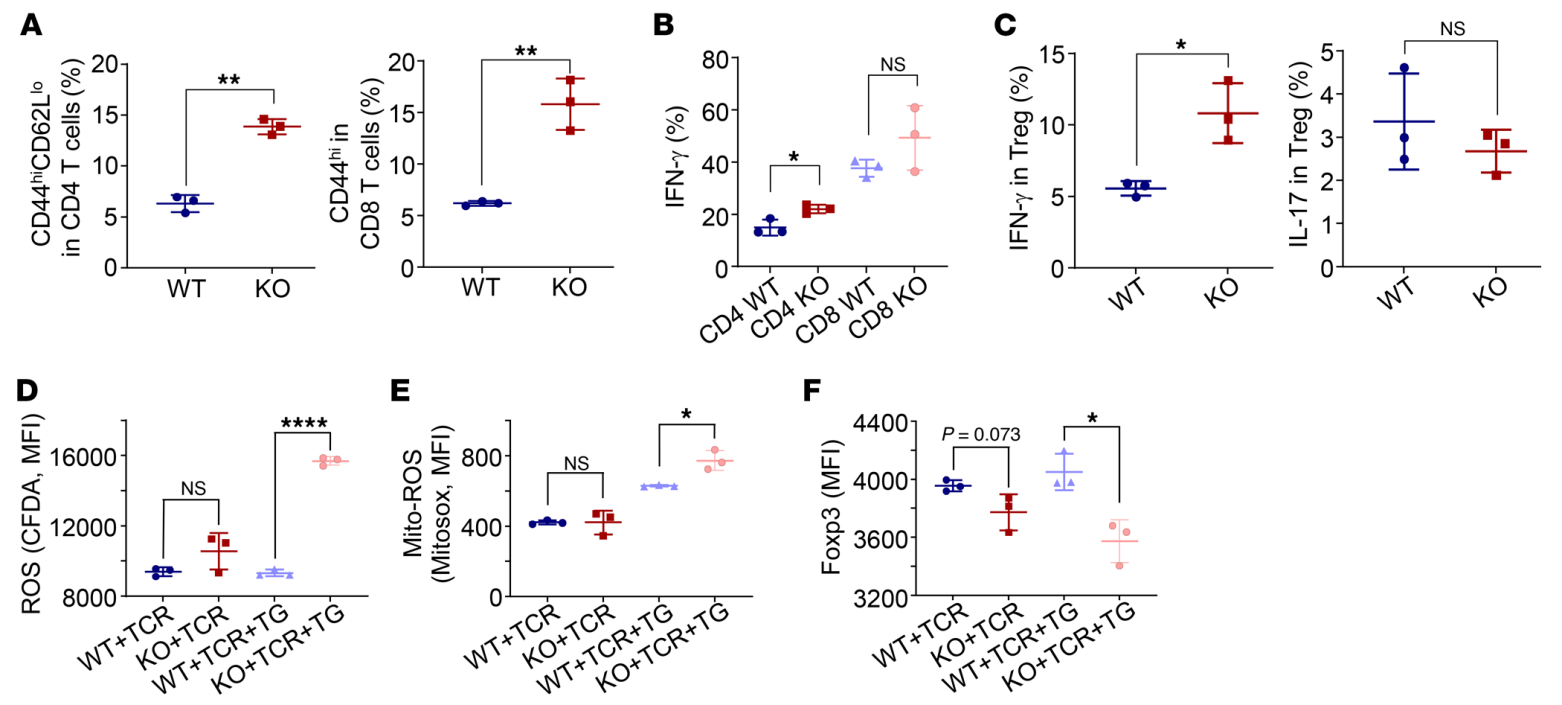
Treg-specific *Ern1*-KO (*Ern1^ΔTreg^*) mice phenocopy *Tmed4^ΔTreg^* mice. (**A** and **B**) Analysis of the proportions of activated (**A**) and IFN-γ–producing (**B**) CD4^+^ and CD8^+^ T cells from spleens of *Ern1^fl/fl^* and *Ern1^ΔTreg^* mice (*n* = 3). (**C**) Analysis of IFN-γ– and IL-17–producing Tregs from spleens from *Ern1^fl/fl^* and *Ern1^ΔTreg^* mice (*n* = 3). (**D**–**F**) Quantitative analysis of the MFI of total ROS (**D**), mitochondrial ROS (**E**), and Foxp3 (**F**) in splenic Tregs from *Ern1^fl/fl^* and *Ern1^ΔTreg^* mice treated with α-CD3/α-CD28 antibodies (TCR), alone or together with 1 μM TG, for 16–20 hours (*n* = 3). Data are presented as the mean ± SEM of biologically independent samples and represent at least 3 independent experiments, each involving 3 mice per group. *P < 0.05, **P < 0.01, and ****P < 0.0001, by 1-way ANOVA with Tukey’s multiple-comparison test (**D**–**F**) and 2-tailed Student’s *t* test.

**Figure 8:**
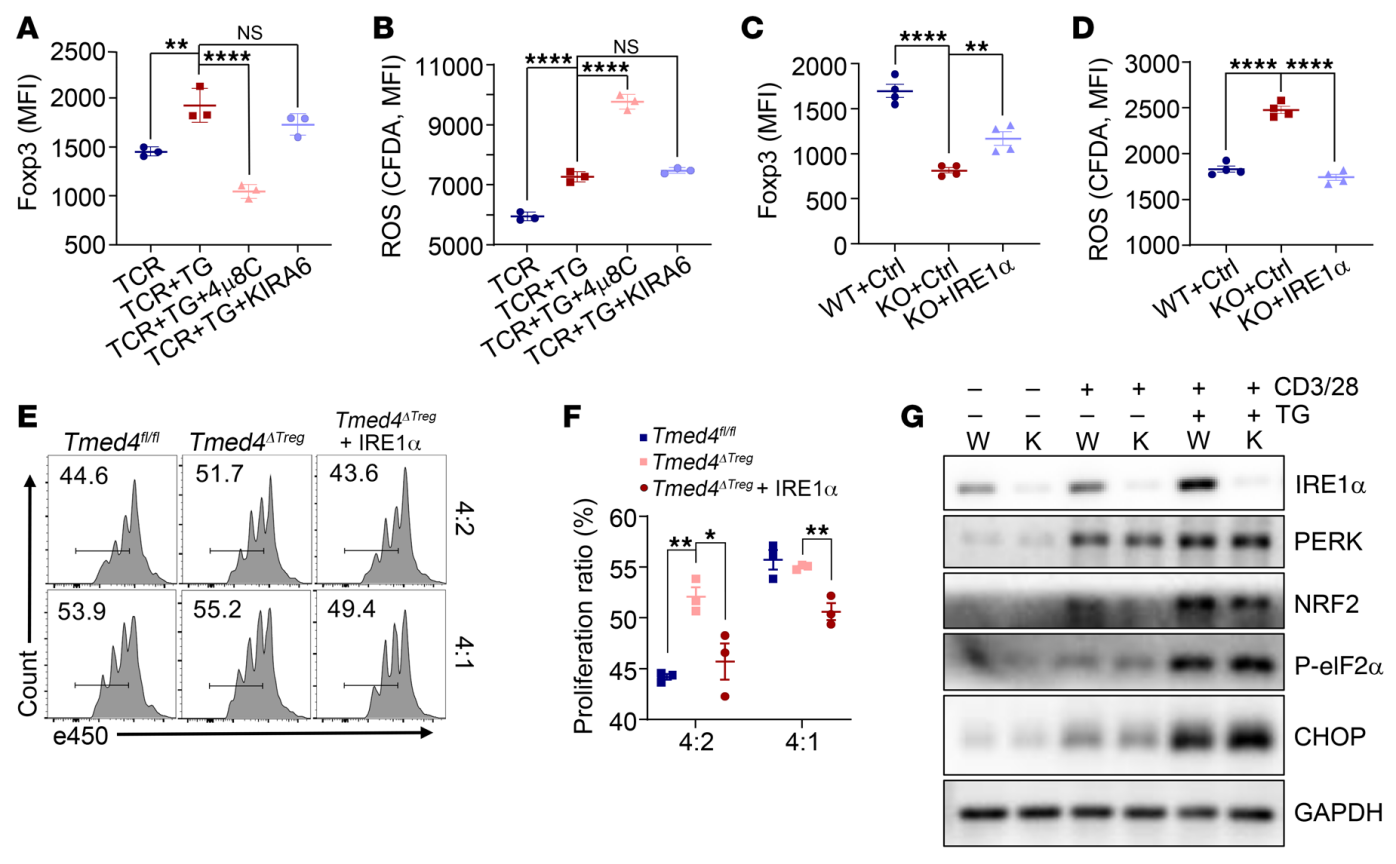
*Tmed4* deficiency in Tregs leads to lower Foxp3 expression and ROS accumulation in an IRE1α/XBP1 axis–dependent manner. (**A** and **B**) Expression levels of Foxp3 (**A**) and cellular ROS (**B**) in WT splenic Tregs treated with α-CD3/α-CD28 antibodies (TCR) and 1 μM TG and the IRE1α inhibitor 4μ8C or KIRA6 for 16–20 hours (*n* = 3). (**C** and **D**) Quantitative analysis of the MFI of Foxp3 expression (**C**) and ROS (**D**) in WT and *Tmed4*-deficient splenic Tregs without (Ctrl) or with induced IRE1α expression. Cells were treated with 1 μM TG for 16–20 hours (*n* = 3). (**E** and **F**) FCM results (**E**) and statistical analysis (**F**) of the in vitro suppressive assay of purified Tregs from spleens of *Tmed4^fl/fl^* and *Tmed4^ΔTreg^* mice or *Tmed4^ΔTreg^* mouse splenic Tregs with forcible expression of IRE1α (*Tmed4^ΔTreg^* + IRE1α), as assessed by the proliferation of activated CD4^+^ T cells at various Treg ratios (Tresp/Treg = 4:2 and 4:1). *n* = 3, detected on day 3. (**G**) Western blot analysis of PERK expression and its downstream proteins in *Ern1^fl/fl^* (W) and *Ern1^ΔTreg^* (K) Tregs treated with DMSO or α-CD3/α-CD28 antibodies, alone or together with 1 μM TG, for 16–20 hours. Data are presented as the mean ± SEM of biologically independent samples and represent at least 3 independent experiments, each involving 3 mice per group. *P < 0.05, **P < 0.01, and ****P < 0.0001, by 1-way ANOVA with Tukey’s multiple-comparison test (**A**–**D** and **F**).

Given that deletion of *Tmed4* leads to a substantial decrease in IRE1α protein levels, along with downstream RNase splicing and kinase activities and lower NRF2 protein levels, we sought to identify the prevailing mechanism governing the regulation of Treg function. To this end, we generated mice with Treg-specific KO of IRE1α (*Ern1^ΔTreg^*). Importantly, we found that *Ern1^ΔTreg^* mice generally phenocopied *Tmed4^ΔTreg^* mice, i.e., IRE1α deficiency in Tregs led to hyperactivation of CD4^+^ and CD8^+^ T cells, as well as the ability to secrete more IFN-γ in CD4^+^ T cells from the KO group (Figure 7, A and B, and Supplemental Figure 9, A and B). Consistent with what we observed in *Tmed4^ΔTreg^* mice, *Ern1*-deficient Tregs gained effector functions and secreted more IFN-γ (Figure 7C and Supplemental Figure 9C). Loss of *Ern1* led to accumulated cellular and mitochondrial ROS compared with WT Tregs (Figure 7, D and E, and Supplemental Figure 9D), along with lower Foxp3 expression levels (Figure 7F and Supplemental Figure 9D). Compared with WT Tregs, *Ern1*-deficient Tregs were defective in their suppressive function (Supplemental Figure 9, E and F). We further dissected whether RNase splicing or kinase activity of IRE1α is more critical in determining the suppressive function of Tregs by applying the IRE1α RNase splicing activity inhibitor 4μ8C or the kinase activity inhibitor KIRA6. We found that 4μ8C, but not KIRA6, treatment of Tregs led to a remarkable reduction in cellular Foxp3 expression and accumulated ROS levels (Figure 8, A and B, and Supplemental Figure 9G). Serving as controls, parallel experiments showed that both inhibitors functioned well (Supplemental Figure 9, H–J).

On the other hand, we assessed whether complementing IRE1α could restore the suppressive function of *Tmed4*-deficient Tregs. To this end, we forcibly induced the expression of IRE1α in *Tmed4*-deficient iTregs by infecting them with Sindbis virus carrying *Ern1* cDNA, followed by confirmation of IRE1α protein expression (Supplemental Figure 10A). Importantly, IRE1α expression restored cellular levels of XBP1s or Foxp3 expression, along with the suppressive function of iTregs, and suppressed ROS levels (Figure 8, C and D, and Supplemental Figure 10B), along with their in vitro suppressive function (Figure 8, E and F).

Loss of *Tmed4* in Tregs partially downregulated phosphorylated eIF2α/NRF2 (p-eIF2α/NRF2), an axis located downstream of PERK (Figure 5L). Therefore, we set out to investigate whether the PERK pathway would independently affect cellular ROS levels and Foxp3 expression. The PERK kinase activity inhibitor GSK2656157, which effectively suppressed the PERK pathway (Supplemental Figure 10C), and concomitant with notably lower NRF2 expression levels, did not cause a significant change in Foxp3 expression levels in Tregs, despite a slight upregulation of ROS levels (Supplemental Figure 10, D–F). To validate the above conclusions more directly, we silenced PERK protein expression in iTregs generated in vitro using an siRNA (Supplemental Figure 10G). Upon PERK silencing, TG-induced ERS still upregulated cellular ROS levels but did not downregulate Foxp3 expression (Supplemental Figure 10, H and I), suggesting that the reduced Foxp3 expression in *Tmed4*-deficient Tregs was not caused by downregulation of the PERK pathway. Interestingly, *Ern1* deficiency did not change the protein abundances of PERK, NRF2, or TMED4 in Tregs under resting or ERS conditions (Figure 8G). Taken together, *Tmed4* deficiency in Tregs led to lower Foxp3 expression and ROS accumulation in an IRE1α/XBP1 axis–dependent manner.

### TMED4 suppresses HRD1/BIP-mediated IRE1α degradation.

**Figure 9:**
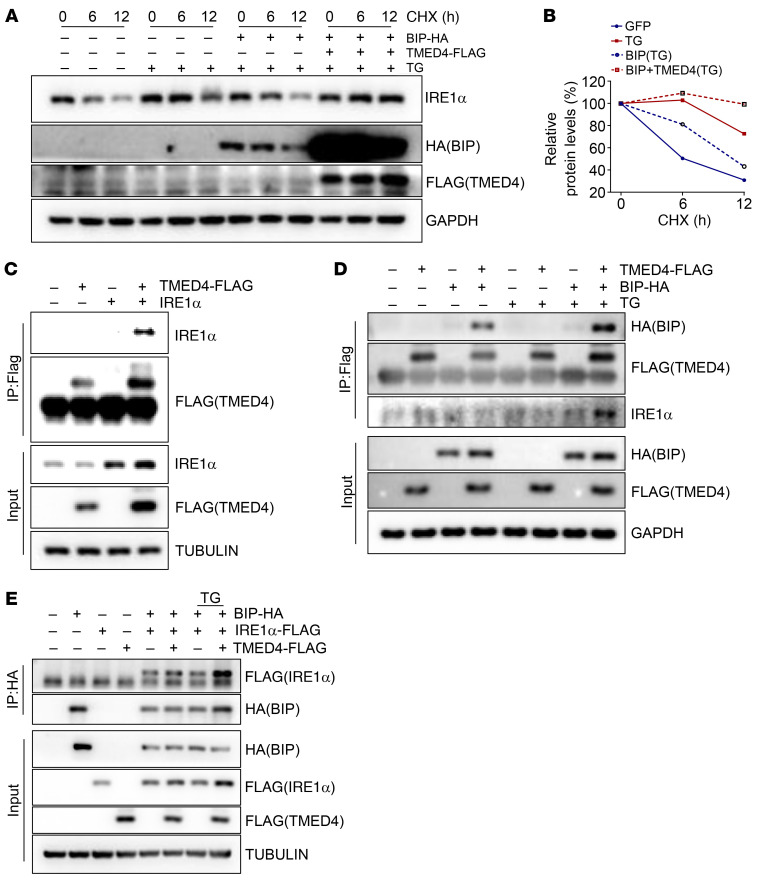
TMED4 suppresses IRE1α degradation in collaboration with BIP. (**A** and **B**) Western blot (**A**) and quantitative analysis (**B**) of IRE1α decay in HEK293T cells transfected or not with TMED4-FLAG and BIP-HA, under treatment with 50 μg/mL CHX with or without 1 μM TG for the indicated durations. (**C** and **D**) Western blot analysis of immunoprecipitation assays on HEK293T cells transfected with TMED4-FLAG and IRE1α (**C**), or BIP-HA (**D**), which were treated or not with 1 μM TG for 6 hours. (**E**) Western blot analysis of immunoprecipitation assays on HEK293T cells transfected with BIP-HA, IRE1α-FLAG, or TMED4-FLAG, which were treated or not with TG for 6 hours. Each blot represents 3 independent experiments with similar results.

**Figure 10:**
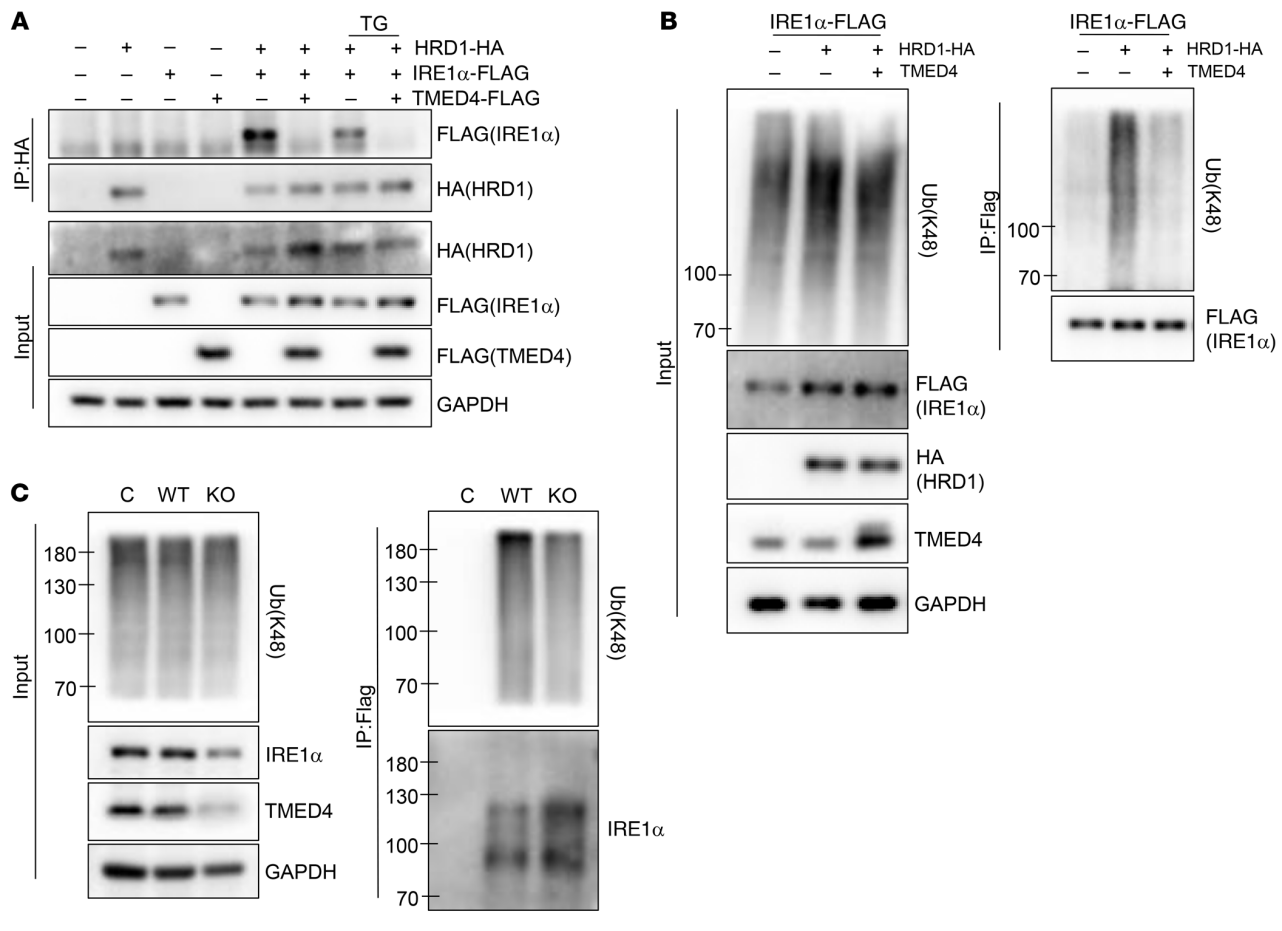
TMED4 suppresses HRD1/BIP-mediated IRE1α degradation. (**A**) WB analysis of immunoprecipitation assays on HEK293T cells transfected with the indicated plasmids, which were treated or not with TG for 6 hours. (**B**) Western blot analysis of IRE1α K48-linked polyubiquitination assays on HEK293T cells transfected with the indicated plasmids. (**C**) Western blot analysis of the IRE1α K48-linked polyubiquitination assay on primary WT and *Tmed4*-deficient iTregs using anti–K48-linked Ub-FLAG beads. Each blot represents 3 independent experiments with similar results.

Given that downregulation of IRE1α protein levels is the leading cause of impaired function of *Tmed4*-deficient Tregs, we sought to identify the regulatory mechanisms through which TMED4 affects the expression of IRE1α protein. When ERS is present, IRE1α forms dimers to activate the downstream ERS response. However, this process can be reversed by BIP overexpression, allowing more IRE1α degradation by the ERAD complex involving synoviolin-1 (SYVN1, also known as HRD1) (12). In 293T cells, TMED4 overexpression inhibited BIP-induced IRE1α degradation (Figure 9, A and B). We further identified that TMED4 interacts with IRE1α and BIP (Figure 9, C and D). Besides, upon induction of ERS, these 3 proteins exhibited enhanced binding with one another (Figure 9, D and E), suggesting that TMED4 facilitated the stabilization and function of IRE1α during ERS, in which BIP might serve as a bridge, mediating the binding of IRE1α to the ERAD complex. TMED4 overexpression alone, regardless of ERS induction, led to IRE1α dissociation from HRD1 (Figure 10A). Moreover, TMED4 remarkably inhibited HRD1-mediated K48 polyubiquitination of IRE1α (Figure 10B). In the in vitro–generated mouse iTregs, *Tmed4* deficiency increased K48-linked ubiquitination of IRE1α (Figure 10C), providing evidence of TMED4-mediated degradation of IRE1α in primary cells. Therefore, the above results implied that TMED4 stabilized IRE1α by blocking the BIP-mediated ERAD system degradation process in Tregs.

## Discussion

In this study, we demonstrate that *Tmed4* deficiency not only led to the loss of ER homeostasis in Tregs, but also resulted in defective mitochondrial activity and excessive accumulation of cellular ROS, which in turn affected cellular metabolic processes and redox signaling homeostasis, causing the downregulation of Foxp3 stability and impaired suppressive functions. Specifically, TMED4 protected Tregs from oxidative stress by maintaining IRE1α protein levels and the equilibrium of the IRE1α/XBP1 axis.

ROS sustains cell survival or the death process by mediating cell signaling pathways and transcription factors via activation of NRF2-associated pathway signals (31). Conventionally, mitochondria have been recognized as the main location of cellular ROS generation. With several recent studies demonstrating that under stressful circumstances, ER luminal ROS levels also increase, and, consequently, the release of calcium and peroxide into the cytosol disrupts the mitochondrial redox reaction (47). In our study, we found that TCR stimulation led to mitochondrial functional defects in Tregs due to *Tmed4* loss, but mitochondrial ROS levels remained unaffected. However, these defects were exacerbated by ERS, indicating delayed ROS production in mitochondria. We suggest that *Tmed4* deficiency may cause ER defects that precede mitochondrial morphological and functional issues in Tregs. Further investigation is needed to understand the complex interplay between ERS and antioxidant responses in immune cell function, particularly Tregs. It is suggested that the regulation of ROS levels in Tregs is not simply binary; an optimal threshold is essential for maintaining their normal and stable functions under stress or pathological conditions.

*Tmed4* deficiency shifts cellular metabolism toward glycolysis over OXPHOS, highlighting the need to investigate its role in the redox metabolic network, particularly in the ER, and its involvement in key substrate dynamics. Glutathione (GSH) deficiency in Tregs, a vital antioxidant for redox homeostasis, produces phenotypes similar to TMED4 deficiency, enhancing cell proliferation and activation, while reducing the expression of Foxp3 and its suppressive function (48). This suggests the potential of TMED4 in regulating ERS responses and the metabolic network supporting antioxidant functions. Further research is needed to clarify whether TMED4 affects the synthesis and degradation of key redox metabolites like GSH, affecting cellular ROS levels and functional stability.

Both the UPR and ERAD processes are evolutionarily conserved quality control systems crucial for maintaining ER homeostasis (49). This balance affects the dynamic activity of IRE1α. In the IRE1α degradation model, BIP acts as a bridge for its interaction with the ERAD complex. The stronger interaction between TMED4 and IRE1α upon BIP overexpression suggests that further investigation is needed to determine if BIP also regulates IRE1α degradation by TMED4, facilitating its dissociation from the ERAD complex and subsequent dimerization to activate downstream UPR signaling pathways. Additionally, the potential role of TMED4 as a chaperone in the UPR, working with BIP to maintain overall ER homeostasis, warrants thorough exploration.

The absence of TMED4 facilitates the conversion of Tregs to Teff cells, enhancing T cell activation and antitumor immunity in the MC38 tumor model. Sustained ERS in the tumor microenvironment hampers the function of tumor-infiltrating T cells. In ovarian cancer, the lack of the IRE1α/XBP1s signaling axis has been shown to boost T cell antitumor activity and prolong host survival (17, 50). Silencing this axis in Tregs by suppressing TMED4 may enhance overall T cell antitumor activity across different tumors. Additionally, CHOP deletion has been linked to increased antitumor activity of CD8^+^ T cells by promoting T-bet expression (51). Our findings indicate that TMED4 deficiency reduced C/EBP-homologous protein (CHOP) levels in Tregs, promoting their conversion to Teff cells and enhancing the activity of other T cell subsets, particularly Th1 and CD8^+^ T cells, although the direct connection remains to be explored. The UPR protein PERK, while not directly involved in our system, also influences CHOP and NRF2 regulation. Understanding the relationship between TMED4 and the PERK regulatory network in managing ROS levels and cellular functions will be essential for clarifying how ERS responses affect Tregs and contribute to antitumor immunity.

In summary, our findings underlie the importance of TMED4 in Treg homeostasis and its role in maintaining the ERS response and mitochondrial activity to avoid the impairment of Treg Foxp3 expression and cellular function caused by excessive accumulation of cellular ROS. TMED4 serves as a rheostat balancing the ERS response and redox signaling pathway. These observations advance our understanding of the role that TMED4 plays in Tregs in alleviating autoimmune diseases and enhancing antitumor immunity.

## Methods

### Sex as a biological variable

We included both male and female sexes in the human samples and study, and sex was not considered as a biological variable. Except for the female chimera experiment, our study examined male mice.

### Mice

CD45.1 mice were purchased from The Jackson Laboratory and provided by Jinke Cheng (SJTU-SM, Shanghai, China). *Tmed4^ΔTreg^* and *Ern1^ΔTreg^* mice were obtained by crossing *Foxp3-YFP-Cre* mice (from The Jackson Laboratory) (52) with *Tmed4^fl/fl^* and *Ern1^fl/fl^* mice that were generated by Shanghai Biomodel Organism Science Technology Development Co., Ltd. and provided by Yong Liu (Wuhan University, Wuhan, China), respectively (53). *Rag1^–/–^* mice were purchased from Cyagen Biosciences. All mice used for experiments were on a C57BL/6 background and 12–16 weeks of age, except for the 8-week-old *Rag1^–/–^* mice used at the time of the experiments. All mice were kept in a specific pathogen–free facility.

### Patients and specimens

Tumor specimens were obtained and processed as previously described (54). Briefly, tumor tissues were dissociated mechanically and enzymatically using Collagenase D (MilliporeSigma) and DNase I (MilliporeSigma) for further isolation of tumor-infiltrating Tregs. Mononuclear cells were then isolated by a Ficoll-Hypaque gradient (GE Healthcare). Isolation of CD4^+^CD25^hi^ Tregs was achieved using flow cytometry (FCM). RNA was quickly extracted with TRIzol reagent (Invitrogen, Thermo Fisher Scientific), reverse-transcribed, and analyzed by quantitative reverse transcription PCR (qRT-PCR).

### Cell culturing and sorting

The HEK293T cell line was purchased from the American Type Culture Collection (ATCC) and cultured in DMEM supplemented with 10% FBS, 40 μM l-glutamine (STEMCELL Technologies), and 1% penicillin-streptomycin. Unless otherwise noted, all lymphocytes were isolated from spleens and pLNs (axillary and inguinal LNs included), from which naive CD4^+^ cells (CD4^+^CD44^lo^CD62L^hi^) or CD4^+^CD25^hi^ Tregs were separated using kits (STEMCELL Technologies) and cultured in RPMI 1640 media supplemented with 10% FBS (Gibco, Thermo Fisher Scientific), 50 μM β-mercaptoethanol (MilliporeSigma), and 1% penicillin-streptomycin. Anti-CD3 (α-CD3) (1 μg/mL) and α-CD28 (1 μg/mL) antibodies were used to stimulate purified Tregs in 96-well, round-bottomed microplates (~2 × 10^5^ cells per well) for FCM, immunoblot (IB), qRT-PCR, and RNA-Seq analysis. For in vitro iTreg generation, 48-well plates coated with α-CD3 (1 μg/mL) and α-CD28 (1 μg/mL) antibodies were used, and naive CD4^+^ cells were isolated from murine splenic cells in the presence of ImmunoCult Mouse Treg Differentiation Supplement (STEMCELL Technologies), 10% FBS, 100 μM HEPES Buffer Solution (STEMCELL Technologies), 40 μM l-Glutamine (STEMCELL Technologies), 100 μM MEM Nonessential Amino Acids Solution (STEMCELL Technologies), 1 mM sodium pyruvate (Thermo Fisher Scientific), 50 μM β-mercaptoethanol (MilliporeSigma), and 1% penicillin-streptomycin in RPMI 1640 medium for 2–6 days.

### Tumor models

Mouse colon cancer (MC38) cells were cultured in RPMI 1640 supplemented with 10% FBS, and these tumor cells were injected s.c. into 8-week-old *Tmed4^fl/fl^* and *Tmed4^fl/fl^* *Foxp3-Cre* mice (5 × 10^5^ cells per mouse). In the adaptive transfer model, purified 2×10^6^ Tregs from *Tmed4^fl/fl^* and *Tmed4^ΔTreg^* donor mice were coinjected s.c. along with 5 × 10^5^ MC38 cells into CD45.1 recipient mice. After tumor inoculation, a second dose of purified Tregs (2 × 10^6^) was injected i.v. 1 week later. Digital calipers were used to measure tumors regularly, and tumor volumes were calculated using the following formula: length × width^2^ × 0.52. A mixture of digestive solution with 0.5 mg/mL collagenase IV (MilliporeSigma) and 200 U/mL DNase I (MilliporeSigma) was used to purify tumor-infiltrating lymphocytes (TILs) from mangled tumor tissue.

### Adoptive BM transfer

A mixture of BM from WT (CD45.1^+^CD45.2^+^) mice and *Tmed4^ΔTreg^* CD45.1^–^CD45.2^+^) mice was transplanted into 6- to 8-week-old mice (CD45.1^+^CD45.2^–^), whose immune system was eliminated by x-ray irradiation. FCM analysis was conducted 8 weeks after BM transfer.

### Induction and evaluation of EAE

To induce acute EAE, 10- to 12-week-old mice were injected s.c. into both flanks of the back regions with 300 μg per mouse of MOG_35–55_ peptide (Met-Glu-Val-Gly-Trp-Tyr-Arg-Ser-Pro-Phe-Ser-Arg-Val-Val-HisLeu-Tyr-Arg-Asn-Gly-Lys, Genemed Synthesis) in CFA (MilliporeSigma) containing 5 mg/mL heat-killed *Mycobacterium tuberculosis* H37Ra (BD Difco). Pertussis toxin (200 ng per mouse; List Biological Laboratories) was also administered to mice in PBS on the day of immunization and 48 hours later. Mice were examined daily for signs of EAE, starting 1 week after immunization. The EAE disease symptoms were graded using the following standard scores: 0 = no abnormality; 1 = tail paralysis; 2 = mild hind limb weakness; 3 = severe hind limb weakness; 4 = complete hind limb paralysis; 5 = quadriplegia or premature state or death. No group information was assigned when evaluating EAE scores.

### Tissue processing and histology

Lung and spinal cords tissues dissected from *Tmed4^fl/fl^* and *Tmed4^ΔTreg^* mice were fixed with 4% paraformaldehyde buffer overnight and washed with 70% ethanol the next day. After washing with 70% ethanol, the tissues were further processed by Wuhan Servicebio Technology Co., Ltd. (paraffin-embedded, sectioned, and stained with H&E and LFB).

### FCM

Single-cell suspensions of lymphocytes were isolated from spleens, LNs, lungs, spinal cords, tumors, or lamina propria from mouse intestines (colon and small intestine). Live cells were selected after staining with BD Fixable Viability Stain. To prepare CNS lymphocytes, the spinal cords and brains were ground on a cell strainer and washed with PBS. After centrifugation at 400*g* for 5 minutes, the cells were resuspended with 37% Percoll (diluted with RPMI complete medium) to 10 mL. The cell suspension was then slowly and gently spread onto a 70% Percoll density gradient at 20°C, 800*g* and centrifuged for 20 minutes. CNS lymphocytes were isolated by collecting the interphase fraction between 30% and 70% Percoll. After intensive washing in PBS, the cells were suspended in RPMI for further use. In FACS buffer (PBS plus 2% FBS plus 2 μM EDTA), cells were blocked by α-CD16/α-CD32 antibodies (1:300) and stained with a primary antibody (1:200) at 4°C for 30 minutes. A fixation and permeabilization kit (BD Biosciences) was used to detect intracellular production of IFN-γ, TNF-α, and IL-17 cytokines after cells were stimulated with stimulation cocktail (Invitrogen, Thermo Fisher Scientific) for 12 hours. And for Foxp3 and ki67 staining, surface marker–stained cells were fixed, permeabilized using True Nuclear staining kit (BioLegend), and labeled with a Foxp3-specific mAb (1:150). FCM data were acquired on an LSRFortessa or LSRFortessa X20 (BD Biosciences) and analyzed with FlowJo_VX software (Tree Star).

### Western blot analysis

Cells were harvested after 2 washes with PBS and lysed in RIPA buffer on ice for 20 minutes to resolve proteins. Cell lysates were centrifuged at 17,000*g* and 4°C for 10 minutes, and the protein concentrations were determined. A 10% SDS-PAGE was then used to resolve the proteins, and the membranes are electrotransfered onto 0.45 μm PVDF (Immobilon-P) membranes. For blocking, 5% skim milk in 1× TBS (10 mM Tris-Cl at pH 7.5 and 150 mM NaCl were added) was used, and the membranes were left at room temperature for 20 minutes. Afterwards, each primary antibody was probed overnight at 4°C on the membranes. A secondary antibody conjugated to HRP (Cell Signaling Technology) was used to detect membranes by ECL after 3 washes with 1× TBS supplemented with 0.05% Tween-20. All primary antibodies were used at a 1:1,000 dilution, with secondary HRP antibodies used at 1:10,000 (diluted in 1% skim milk).

### In vitro Treg suppression assay

CD4^+^CD25^+^ Tregs were purified from the spleens of 8- to 12-week-old *Tmed4^fl/fl^* and *Tmed4^ΔTreg^* mice. CD4^+^CD25^–^ T cells were purified from WT mice and labeled with e450 (eBioscience). CD4^+^CD25^–^ T cells with e450-labeled responder cells (1 × 10^5^) and Tregs (at ratios of 1:2, 1:1, and 1:0.5) were cocultured in 96-well plates coated with α-CD3 (1 μg/mL) and α-CD28 (1 μg/mL) antibodies for 3 days. In addition, in cases in which Tregs needed to be pretreated before the Treg suppression assay, cells were stimulated with α-CD3/α-CD28 antibodies (1 μg/mL), IL-2 (200 U/mL) (Peprotech) in complete medium (containing 10% FBS and 1% penicillin-streptomycin) supplemented with drug for the indicated durations.

### In vitro cell treatments

For the FOXP3 and IRE1α half-life test, CHX (Selleck Chemicals) was used at 1 μg/mL and 50 μg/mL on primary Tregs or 293T cells for the indicated durations. For ERS induction, primary Tregs or 293T cells were pretreated with 1 μM TG (MilliporeSigma) for 6–12 hours. 4μ8C, KIRA6, and GSK2656157 were purchased from Selleck Chemicals and used at 10 μM in the inhibitory experiments. Acetylcysteine (NAC) and sulforaphane were purchased from Selleck Chemicals and used at 1 mM and 10 μM, respectively, in the in vitro cell function rescue experiments. For the Treg phenotype rescue experiments in the MC38 tumor model, the inoculated Tregs were pretreated with NAC for 12 hours at 1 mM.

### qRT-PCR and RNA-Seq

TRIzol reagent (Invitrogen, Thermo Fisher Scientific) was used to extract total RNA from Tregs purified from *Tmed4^fl/fl^* and *Tmed4^ΔTreg^* mice, and RNA was quantified with NanoDrop (Thermo Fisher Scientific). Total RNA (1 μg) was reverse transcribed using a reverse transcription kit (Yeasen). Gene-specific primers (SYBR Green, Yeasen) were used to quantify the indicated genes from purified cDNA. Gene expression levels in the same sample were normalized to *Actb* or *Gapdh* mRNA levels. Relative quantitation of target cDNA was determined by the formula 2^ΔCt^, with ΔCt denoting fold increases above the set control value. The primer sequences are listed in Supplemental Table 1. Total RNA (1 μg) was used for RNA-Seq with 3 biological replicates per genotype, and RNA-Seq was performed using the Illumina system with 150-base paired-end reads. A default alignment was performed using hisat2 with the mouse reference genome (mm10) as the reference. Genes with differential expression were identified and normalized by DESeq2 (|log_2_(fold change)| ≥1.5; *P* < 0.05). R package Cluster Profiler was used to perform GO and KEGG enrichment analyses, where the DEGs identified as described above were supplied as the input for genes by function and by GO enrichment and KEGG enrichment, respectively. Gene set enrichment analysis (GSEA) analysis was done using GSEA software (version 4.1.0; https://www.gsea-msigdb.org/gsea/downloads.jsp). After loading the expression data formats and phenotype data formats, GSEA was run using default parameters.

### Plasmid and transfections

TMED4-overexpressing human vector (TMED4 and TMED4-FLAG in pCDH-CMV-MCS-EF1-Puro) was generated by Azenta Life Sciences. Human plasmids with overexpression of BIP-HA (in pCDH or pCDNA3.0 vector), IRE1α-FLAG (pCDNA3.0), and HRD1-HA (pCDNA3.0) were provided by Zhaoyuan Hou (SJTU-SM, Shanghai, China). The IRE1α-overexpressing human plasmid was provided by Bin Li (SJTU-SM, Shanghai, China). One 6-well plate containing 2 × 10^6^ to 3 × 10^6^ 293T cells was transfected with 1 μg plasmids each and polyethyleneimine (PEI) (Polyscience). After 24–48 hours, cells were treated or not with fresh media with 1 μM TG for 5 hours, followed by cell lysis for further use.

### siRNA knockdown

iTregs were transfected by electroporation as described previously (54) with 50 nM mouse PERK or nontargeting control siRNA. Electroporation was carried out using the Mouse T cell Nucleofector Kit (Lonza). Transfected cells were lysed 48–72 hours after transfection for further experiments. The sequences specific for PERK are shown in Supplemental Table 2.

### Seahorse assays

Tregs were stimulated for 24 hours with α-CD3 and α-CD28 antibodies and attached to culture plates with CellTak (Corning) at a density of 3 × 10^5^ cells per well. The OCR and ECAR of T cells were measured on the Seahorse Biosciences XF96 Extracellular Flux Analyzer (Seahorse Biosciences). The following are the concentrations of compounds injected: 10 mM glucose, 100 μM oligomycin, 100 μM FCCP, 50 μM rotenone/antimycin A (Agilent Technologies).

### T cell transfer colitis model

*Rag1*-deficient mice were injected with naive CD4^+^CD25^–^CD45RB^hi^ splenic T cells (0.3 million cells/mouse) isolated from B6.SJL (CD45.1^+^) mice alone or in combination with CD4^+^CD25^+^ Tregs from *Tmed4^fl/fl^* or *Tmed4^ΔTreg^* (CD45.2^+^) mice (0.1 million cells/mouse). Mice were monitored weekly for weight loss for 6 weeks, after which mice were sacrificed for further analysis.

### iTreg virus infection model

Naive CD4^+^ T cells were sorted from *Tmed4^fl/fl^* or *Tmed4^ΔTreg^* mice and transformed into iTregs as described in *Cell culturing and sorting*. The Sindbis expression system was provided by Peng Zhang (SJTU-SM, Shanghai, China). iTregs were infected with GFP control or IRE1α-GFP Sindbis concentrated virus with 5 μg/mL polybrene at 900*g*, 32°C for 2 hours, and the fresh induction differentiation medium was replaced after 6 hours. After 24 hours, GFP^+^ cells were sorted for use in experiments.

### Statistics

A Pearson’s correlation coefficient was used to analyze the correlation between genes in the human data shown in Supplemental Figures 1 and 6. Statistical analyses were conducted using a 2-tailed Student’s *t* test or 1-way ANOVA with Tukey’s multiple-comparison test. Data are shown as the mean ± SEM. Statistical analysis was performed with GraphPad Prism 7.0 (GraphPad Software). A *P* value of less than 0.05 was considered statistically significant.

### Study approval

All patients with colon or stomach adenocarcinoma provided informed consent prior to inclusion in the study, and all procedures were approved by the ethics committee of Ruijin Hospital at SJTU-SM (2019-186). All mouse experimental procedures were conducted with the approval of the Department of Laboratory Animal Science (DLAS) of SJTU-SM.

### Data availability

#### Lead contact.

Further information and requests for resources and reagents should be directed to and will be fulfilled by the corresponding author Xuefeng Wu.

#### Materials availability.

This study did not generate new unique reagents (see Supplemental Table 3 for the list of antibodies and reagents used).

#### Data availability.

RNA-Seq data are available in the Sequence Read Archive (SRA) under accession number PRJNA1025114. This work involved no original code. All other data are reported in the Supporting Data Values file. Any additional information required to reanalyze the data reported in this paper is available from the lead contact upon request.

## Author contributions

ZJ and HW designed and performed the experiments, prepared the figures, and wrote the manuscript. Xiaoxia Wang designed experiments and helped analyze data. HD analyzed TCGA database. YT, J Li, XL, J Liu, JN, EJW, HX, CG, and Xinyu Wang contributed to part of the experiments. KZ and PZ helped with the Sindbis experiments. ZH, YL, and ZW provided technical support, discussions, and advice. BS, Bo Li, YH, and Bin Li supervised the research. X Wu designed experiments, supervised the research, and wrote the manuscript.

## Supplementary Material

Supplemental data

Unedited blot and gel images

Supporting data values

## Version 1 10/31/2024

In-Press Preview

## Version 2 01/02/2025

Electronic publication

## Version 3 01/08/2025

Corrected affiliations for Huizi Wang

## Version 4 06/09/2026

Corrected Supporting Data Values file for Figure 2K.
