# Supplementary material for: TMED4 facilitates regulatory T cell suppressive function via ROS homeostasis in tumor and autoimmune mouse models
Source: J Clin Invest. 2024 Oct 31;135(1):e179874. doi: 10.1172/JCI179874 (PMC11684806; doi:10.1172/JCI179874)
Supplement: Supplemental data [file jci-135-179874-s011.pdf]

This PDF file contains: Supplementary Figures S1 to S10 and Tables 1-3.

Supplementary Figures:

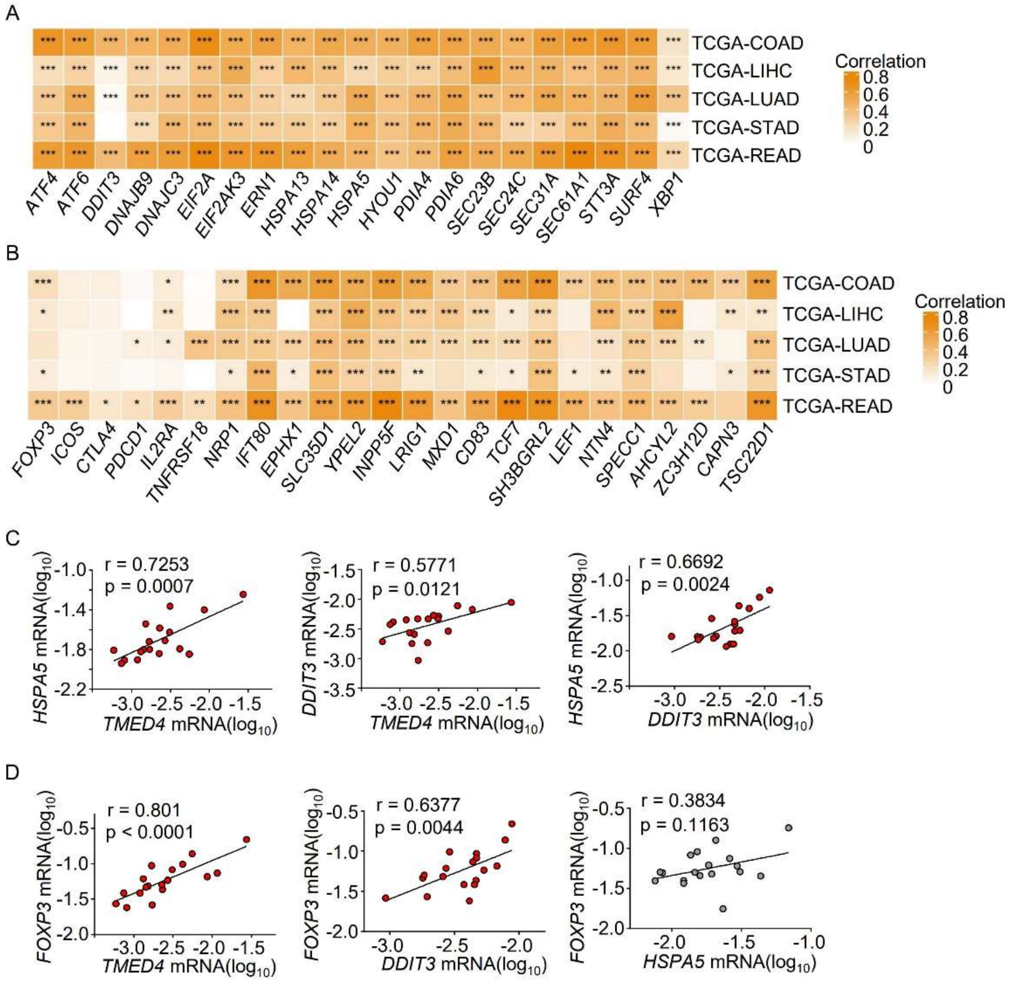

Figure S1. TMED4 positively correlates with ER stress responses and FOXP3 expression in tumor-infiltrating Treg cells.

(A and B) The heatmap showing the Pearson correlation coefficients between the expression values of ER stress response-associated genes (A)/Treg signatures (B) and *TMED4* from The Cancer Genome Atlas (TCGA)-colon adenocarcinoma (COAD)/-liver hepatocellular carcinoma (LIHC)/-lung adenocarcinoma (LUAD)/-stomach adenocarcinoma (STAD)/-rectum adenocarcinoma (READ) data. *p < 0.05, **p < 0.01, and ***p < 0.001.

(C and D) Pair-wise analyses for human colorectal, gastric and renal cancer associated Treg cells (n = 18 total). Expression of genes linked with ER stress responses and *TMED4* (C). *FOXP3* versus genes associated with ER stress responses (D) ‘Pearson’s rank correlation test, Pearson coefficient (r) with p value (two-tailed), 95% confidence intervals for all correlation analyses. n values correspond to biologically independent samples.

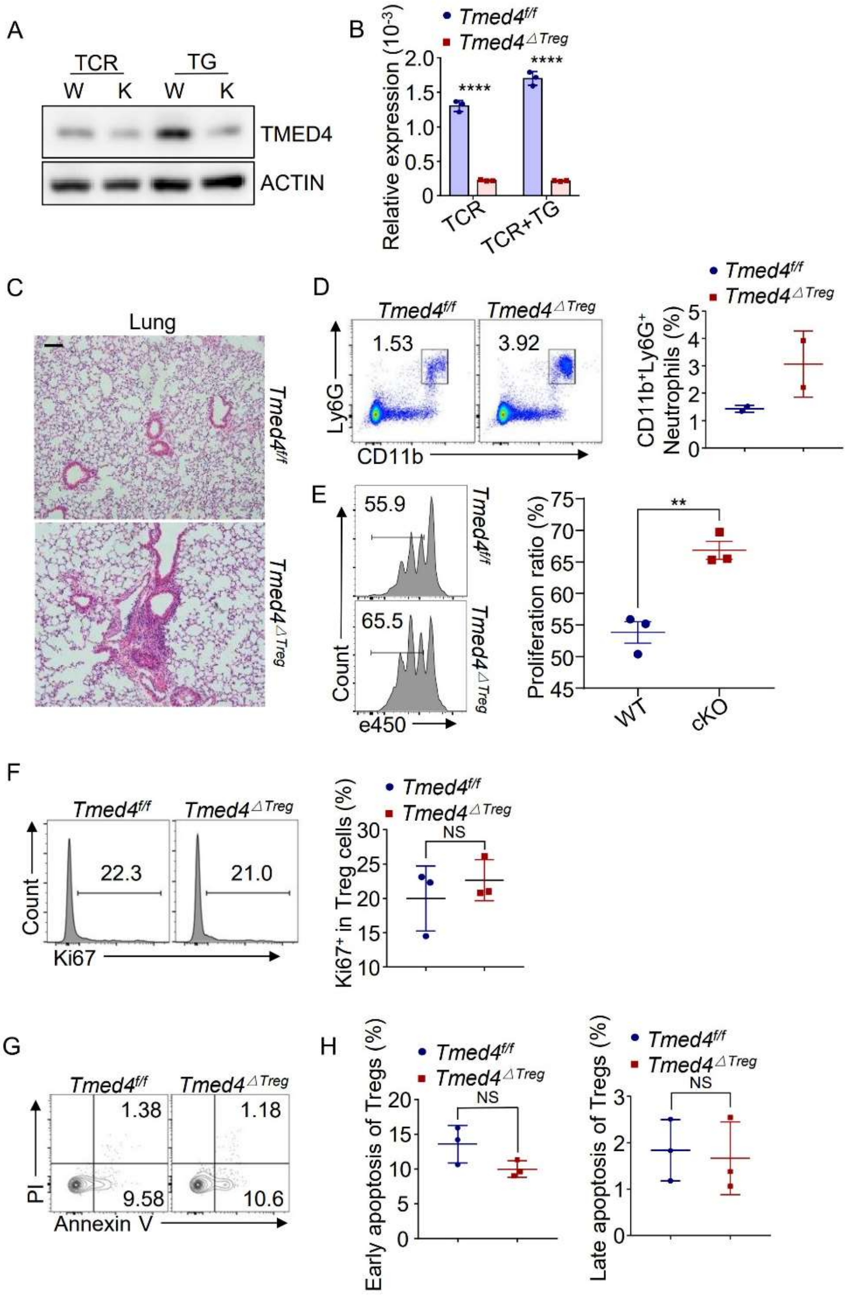

Figure S2. *Tmed4* deficiency impairs Treg cells' signature profiles in a cell intrinsic manner.

(A and B) The TMED4 protein (A) and mRNA (B) knockout efficiency of purified Treg cells ($CD4^+CD25^+$) by western blot(A) and qRT-PCR(B) stimulated with α-CD3/28 and 1 μM thapsigargin (TG) (n = 3 for qRT-PCR).

(C) H&E staining of lung tissue. Scale bar: 100 μm.

(D) FCM plots (D, left) and analysis (D, right) of neutrophil ($CD11b^+Ly6G^+$) abundance among splenocytes from *Tmed4*$^{f/f}$ and *Tmed4*$^{\Delta Treg}$ mice (n = 2).

(E) FCM plots (E, left) and analysis (E, right) of in vitro proliferation assay of Treg cells from *Tmed4*$^{f/f}$ and *Tmed4*$^{\Delta Treg}$ mice (n = 3).

(F) FCM levels (F, left) and statistical analysis (F, right) of $Ki67^+$ percentages of Treg cells from *Tmed4*$^{f/f}$ and *Tmed4*$^{\Delta Treg}$ mice (n = 3).

(G and H) FCM levels (G) and statistical analysis (H) of apoptosis levels of splenic Treg cells from *Tmed4*$^{f/f}$ and *Tmed4*$^{\Delta Treg}$ mice (n = 3). Annexin V^+ PI^- represents the

early stage and Annexin V^+ PI^+ represents the later stage.
Data are presented as mean ± SEM of biologically independent samples and each represents at least 3 independent experiments, each involving 2-4 mice per group. NS, not significant, *$p < 0.05$, **$p < 0.01$, ***$p < 0.001$, and ****$p < 0.0001$, by two-tailed Student's t test.

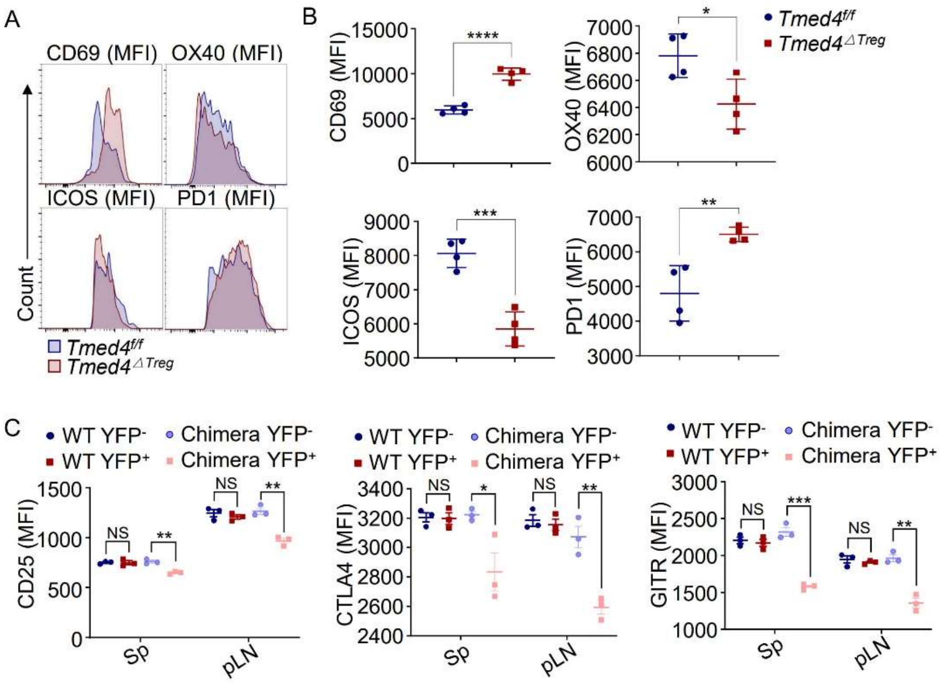

Figure S3. *Tmed4* deficiency impairs Treg cells' signature profiles in a cell intrinsic manner.

(A and B) FCM levels (A) and statistical analysis (B) of CD69, OX40, ICOS and PD1 MFI of *Tmed4*$^{f/f}$ and *Tmed4*$^{\Delta Treg}$ mice (n = 4).

(C) FCM analysis of CD25 (C, left), CTLA4 (C, middle) and GITR (C, right) MFI between YFP^{+} and YFP^{-} Treg cells in female WT and chimera mice (n = 3).

Data are presented as mean ± SEM of biologically independent samples and each represents at least 3 independent experiments, each involving 4 mice per group. NS, not significant, *p < 0.05, **p < 0.01, ***p < 0.001, and ****p < 0.0001, by two-tailed Student's t test.

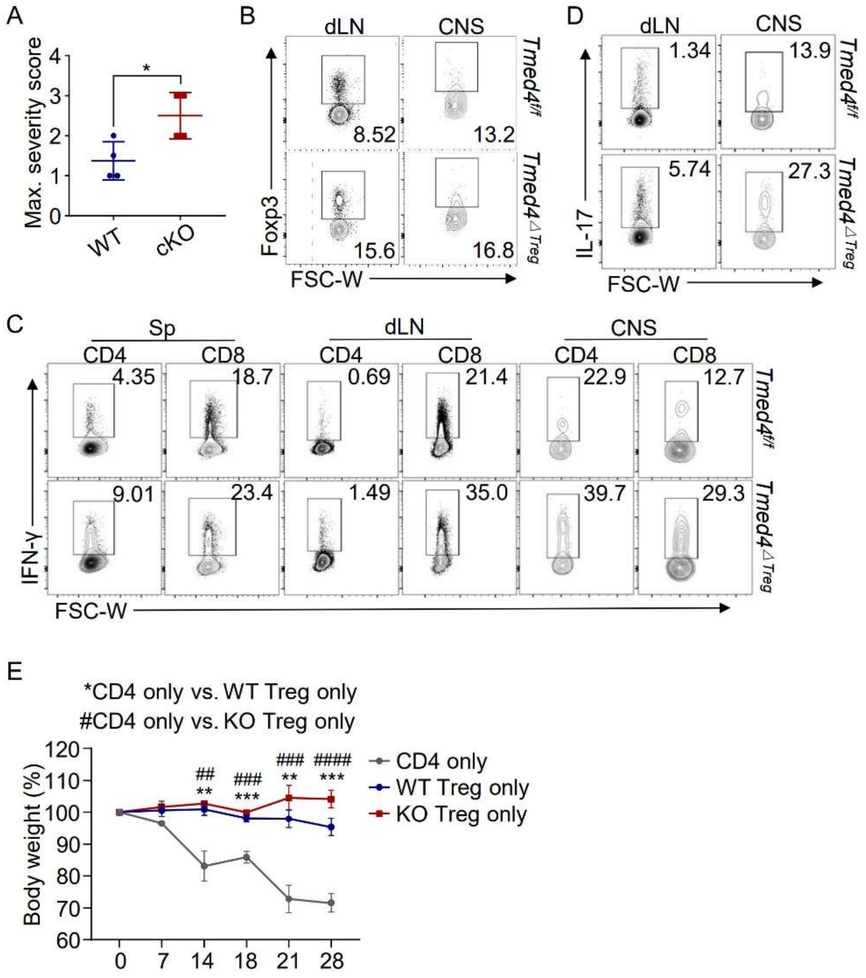

Figure S4. Loss of *Tmed4* in Treg cells leads to more exacerbated inflammatory phenotype in mice.

(A) Maximal severity score of diseased *Tmed4*$^{f/f}$ and *Tmed4*$^{\Delta Treg}$ mice (n = 4).

(B-D) Flow cytometry (FCM) plots of Foxp3(B) IFN-γ(C) and IL-17(D) from spleen, dLN (draining lymph node), and CNS (central nervous system) of diseased *Tmed4*$^{f/f}$ and *Tmed4*$^{\Delta Treg}$ mice.

(E) The curve of body weight percentage. Injections of $CD4^{+}CD45RB^{hi}CD25^{lo}$ naïve T cells or Treg cells isolated from *Tmed4*$^{f/f}$ and *Tmed4*$^{\Delta Treg}$ mice were made in *Rag1*$^{-/-}$ mice alone. The body weight is presented relative to the initial weight in each case (n = 4). Data are presented as mean ± SEM of biologically independent samples and each represents at least 2 independent experiments, each involving 4 mice per group. NS, not significant, *$p < 0.05$, **$p < 0.01$ or ##$p < 0.01$, ***$p < 0.001$ or ###$p < 0.001$, and ****$p < 0.0001$ or ####$p < 0.0001$, by one-way analysis of ANOVA with Tukey's multiple-comparison test (E) and two-tailed Student's t test.

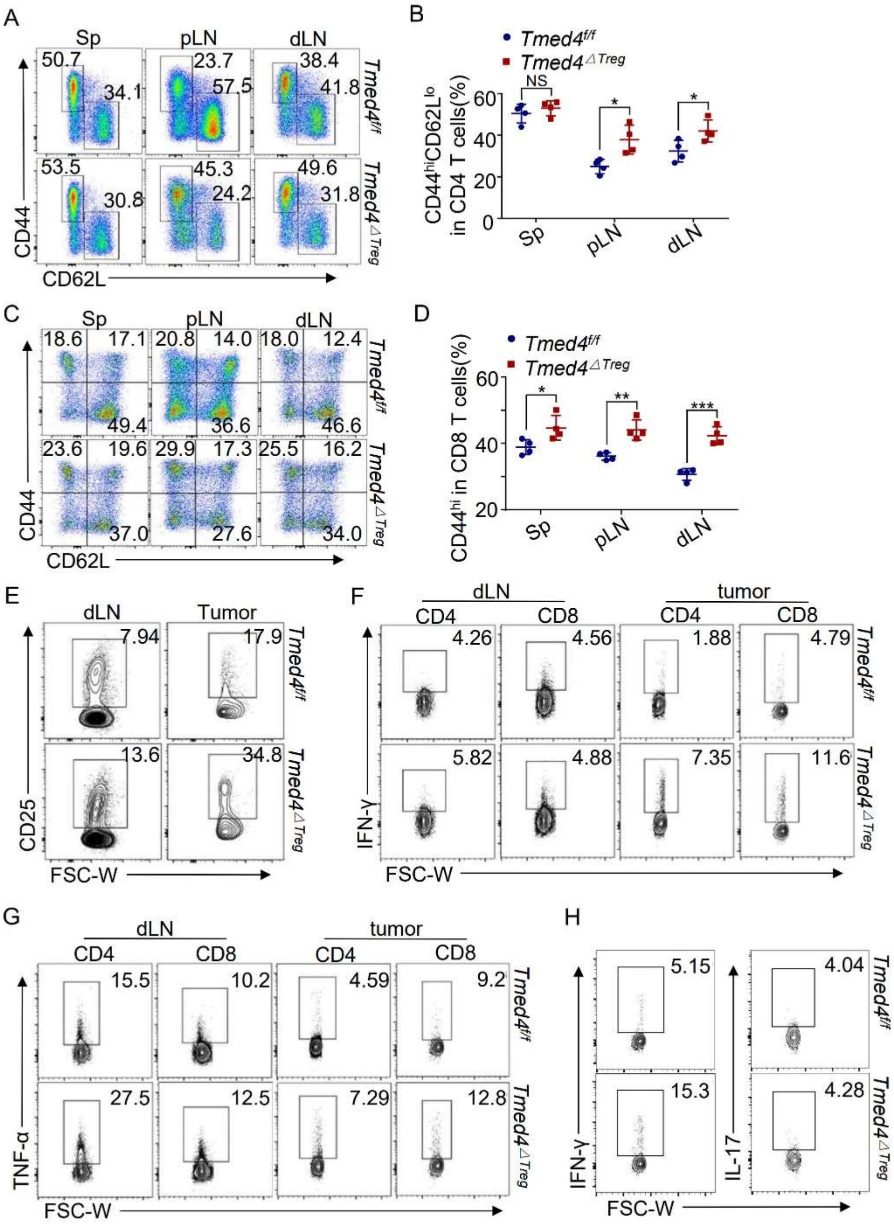

Figure S5. Loss of *Tmed4* in Treg cells leads to boosted anti-tumor immunity in mice. (A-D) FCM plots and analysis of effector $CD4^+$ (A and B) and $CD8^+$ (C and D) T cells isolated from spleen, pLN (peripheral lymph node), and dLN (draining lymph node) in tumor bearing *Tmed4*$^{f/f}$ and *Tmed4*$^{\Delta Treg}$ mice (n = 4).

(E) FCM plots of tumor-infiltrating Treg cells ($CD4^+CD25^+$) from dLN and tumor of MC38 tumor bearing mice (n = 4).

(F and G) FCM plots of IFN-γ (F) and TNFα (G) production from $CD4^+$ and $CD8^+$ T cells in dLN and tumor of *Tmed4*$^{f/f}$ and *Tmed4*$^{\Delta Treg}$ tumor bearing mice (n = 4).

(H) FCM plots of tumor infiltrating Treg cells that producing IFN-γ and IL-17 from tumor bearing *Tmed4*$^{f/f}$ and *Tmed4*$^{\Delta Treg}$ mice.

Data are presented as mean ± SEM of biologically independent samples and represents at least 3 independent experiments, each involving 4 mice per group. NS, not significant, *p < 0.05, **p < 0.01, ***p < 0.001, and ****p < 0.0001, by two-tailed Student's t test.

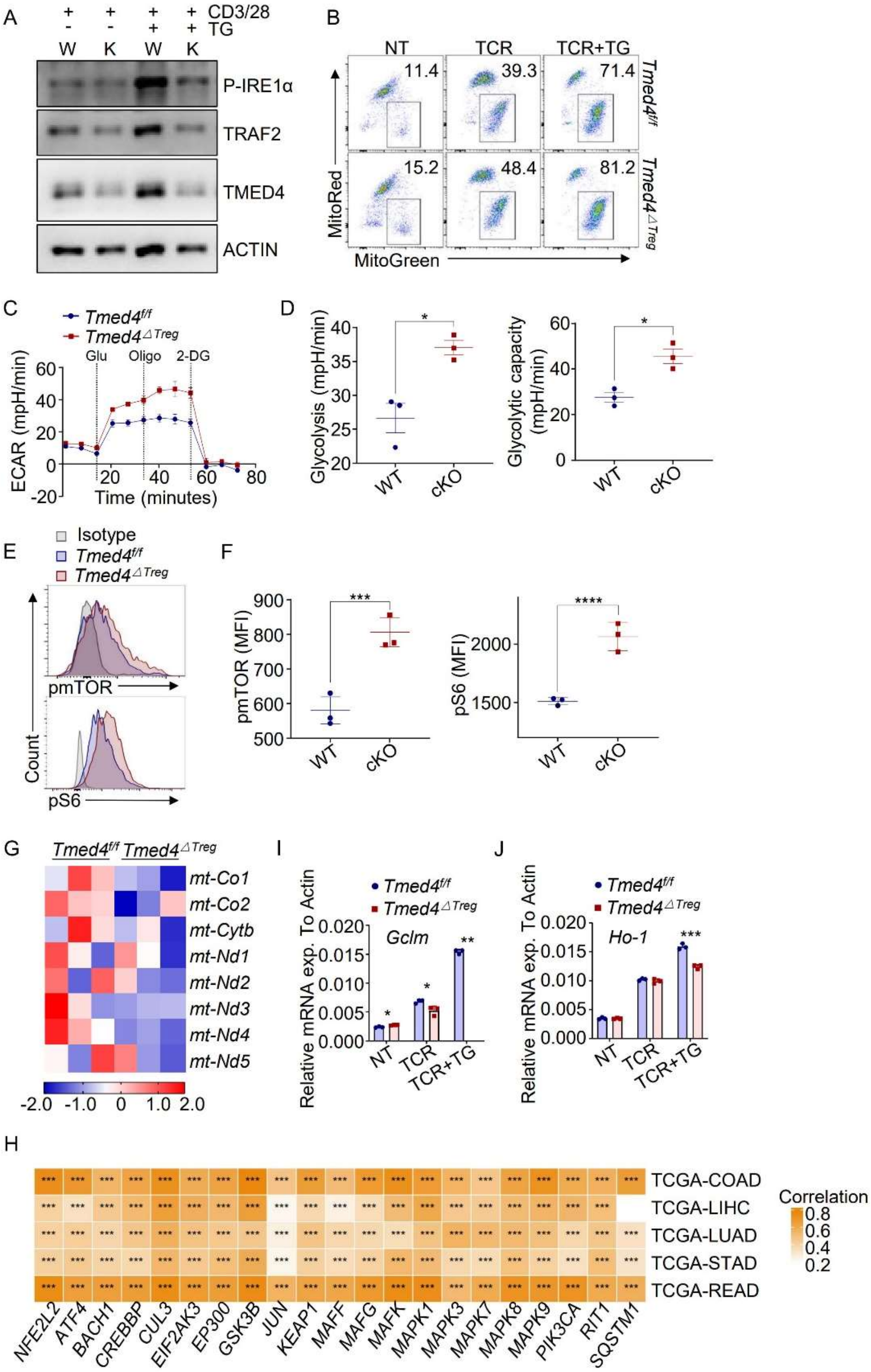

Figure S6. *Tmed4*-deficiency leads to impaired ER stress response, mitochondria integrity in Treg cells.

(A) WB analysis of IRE1α kinase activity-related proteins in WT and *Tmed4*-deficient Treg cells treated with α-CD3/28 alone or together with 1 μM thapsigargin (TG) for 16-20 h.

(B) FCM analysis of mitochondria by staining with Mito-Tracker (MitoRed and MitoGreen) in WT and *Tmed4*-deficient splenic Treg cells treated with α-CD3/28 (TCR) alone or together with thapsigargin (TG) for 16-20 h (n = 2).

(C and D) The curve (C) and quantitative analysis (D) of Extracellular acidification rate (ECAR) of WT and *Tmed4*-deficient Treg cells stimulated with α-CD3/28 for 16-20 h (n = 3).
(E and F) The FCM levels (E) and statistical analysis (F) of phosphorylated-mTOR and S6 between WT and *Tmed4*-deficient Tregs isolated from spleen (n = 3).
(G) Heatmap of gene clusters of components of mitochondrial complex genes between WT and *Tmed4*-deficient Treg cells. Red and blue represent relatively higher and lower levels of expression of indicated genes, respectively. The colors indicate the value of log2 fold change (n = 3).
(H) The heatmap showing the Pearson correlation coefficients between the expression values of antioxidant response-associated genes and *TMED4* from The Cancer Genome Atlas (TCGA)-colon adenocarcinoma (COAD)/-liver hepatocellular carcinoma (LIHC)/-lung adenocarcinoma (LUAD)/-stomach adenocarcinoma (STAD)/-rectum adenocarcinoma (READ) data.
(I and J) Relative mRNA expression of *Gclm* (I) and *Ho-1* (J) in WT and *Tmed4*-deficient Treg cells under resting or with α-CD3/28 (TCR) alone or together with thapsigargin (TG) for 16-20 h (n = 3).
Data are presented as mean ± SEM of biologically independent samples and represents at least 3 independent experiments, each involving 2-3 mice per group. NS, not significant, *p < 0.05, **p < 0.01, ***p < 0.001, and ****p < 0.0001, by two-tailed Student’s t test.

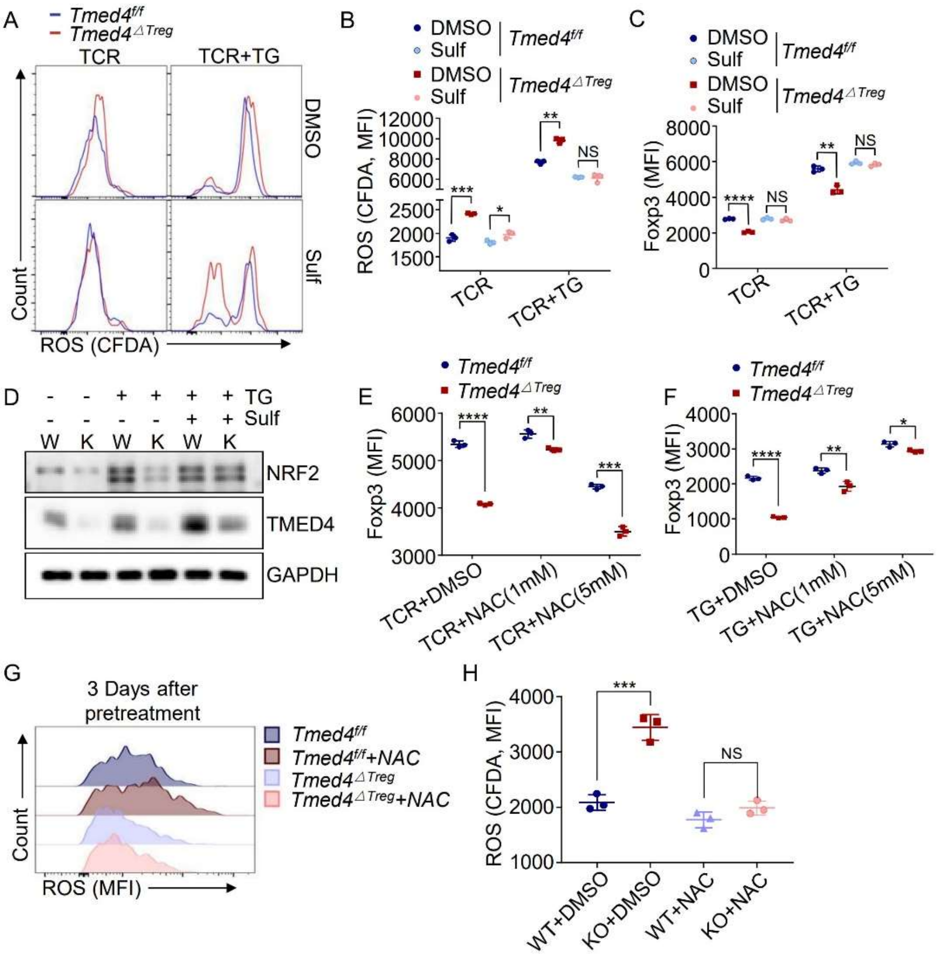

Figure S7. ROS scavenger or NRF2 inducer restores Foxp3 expression and suppressive function of *Tmed4*-deficient Treg cells

(A-C) Cellular ROS levels (A) and their quantitative analysis of MFI (B) and Foxp3 MFI (C) in splenic WT and *Tmed4*-deficient Treg cells treated with TCR alone or together with thapsigargin (TG) for 12 h in the presence or absence of Sulf (Sulforaphane) (n = 3).

(D) WB analysis of NRF2 and TMED4 protein levels in WT (W) and cKO (K) Treg cells treated as above.

(E and F) The Foxp3 expression levels of splenic WT Treg cells treated with TCR (E) alone or together with thapsigargin (TG) (F) for 12 h in the presence or absence of NAC (N-acetylcysteine) at the concentration of 1 mM or 5 mM (n = 3).

(G and H) The FCM levels (G) and statistical analysis (H) of cellular total ROS 3 days after NAC pretreatment (n = 3).

Data are presented as mean ± SEM of biologically independent samples and represents at least 3 independent experiments, each involving 3 mice per group. NS, not significant, *p < 0.05, **p < 0.01, ***p < 0.001, and ****p < 0.0001, by two-tailed Student's t test.

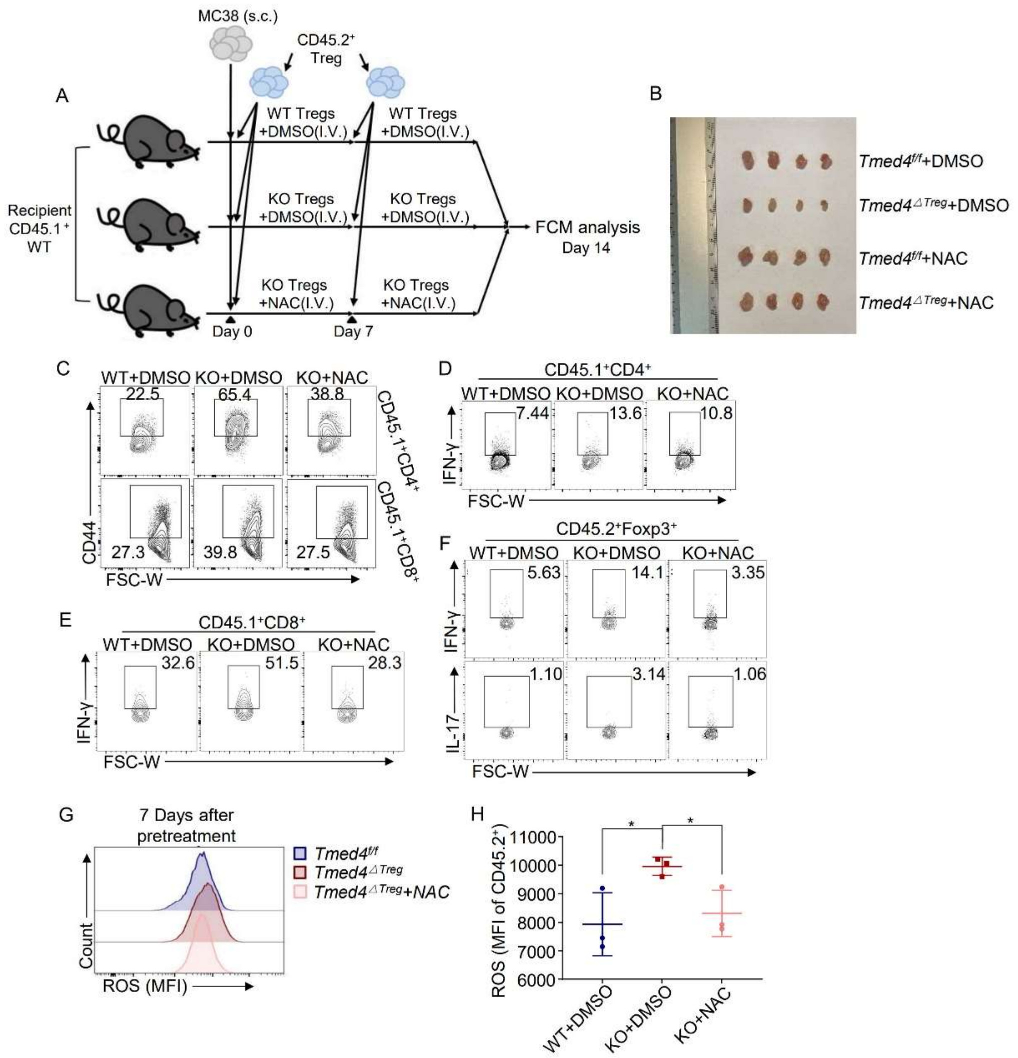

Figure S8. *Tmed4*-deficiency in Treg cells led to lower Foxp3 expression and ROS accumulation in an IRE1α-XBP1 axis dependent manner

(A) The schematic diagram of tumor model on CD45.1^{+} recipient mice with CD45.2^{+} donor Treg adoptive transfer.

(B) The image of tumor growth in CD45.1^{+} mice injected (s.c.) with MC38 cells together with WT or *Tmed4*-deficient Treg cells and then on day 7 intravenously (i.v.) treated with WT or *Tmed4*-deficient Treg cells again (Treg cells were pretreated with α-CD3/28 for 12 h in the presence or absence of NAC).

(C) The FCM plots of T cell activation of host CD45.1^{+} T cells with WT or *Tmed4*-deficient Treg cells pretreated with α-CD3/28 for 12 h in the presence or absence of NAC.

(D and E) The FCM plots of IFN-γ-producing CD45.1^{+}CD4^{+} (D) and CD45.1^{+}CD8^{+} (E) T cells with WT or *Tmed4*-deficient Treg cells pretreated with α-CD3/28 for 12 h in the presence or absence of NAC.

(F) The FCM plots of IFN-γ and IL-17-producing CD45.2^{+} Treg cells pretreated with α-CD3/28 for 12 h in the presence or absence of NAC.

(G and H) The FCM levels (G) and statistical analysis (H) of cellular total ROS 7 days after NAC pretreatment (n = 3).

Data are presented as mean ± SEM of biologically independent samples and represents at least 2 independent experiments, each involving 3 mice per group. NS, not significant, *P < 0.05, **P < 0.01, ***P < 0.001, and ****P < 0.0001, by one-way analysis of

ANOVA with Tukey’s multiple-comparison test (H).

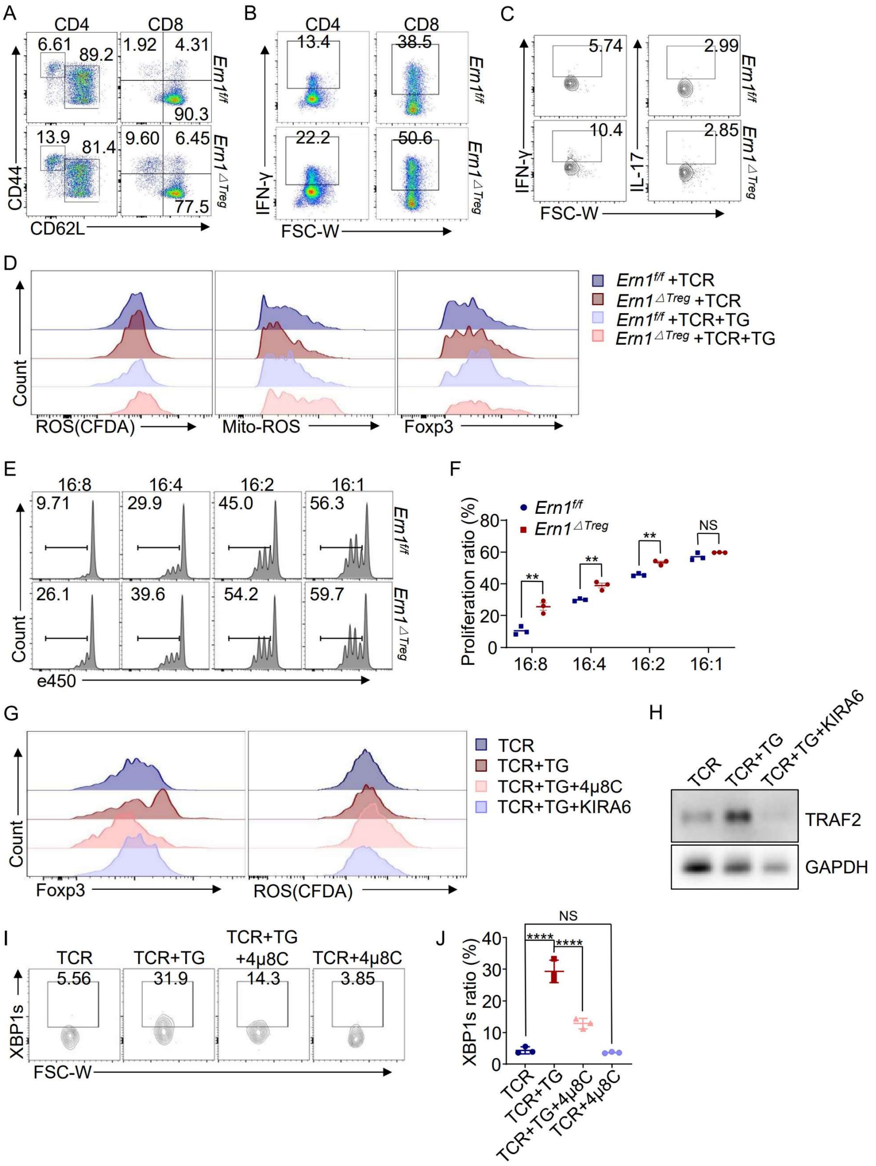

Figure S9. *Tmed4*-deficiency in Treg cells led to lower Foxp3 expression and ROS accumulation in an IRE1α-XBP1 axis dependent manner

(A and B) The FCM plots of T cell activation (A) and IFN-γ-producing (B) T cells isolated from spleens in *Ern1*$^{f/f}$ and *Ern1*$^{\Delta Treg}$ mice.

(C) The FCM plots of IFN-γ and IL-17-producing Treg cells isolated from spleens in *Ern1*$^{f/f}$ and *Ern1*$^{\Delta Treg}$ mice.

(D) Levels of cellular total ROS (D, left), mitochondrial ROS (D, middle) and Foxp3 (D, right) of *Ern1*$^{f/f}$ and *Ern1*$^{\Delta Treg}$ splenic Treg cells treated with α-CD3/28 (TCR) alone or together with thapsigargin (TG) for 16-20 h (n = 3).

(E and F) FCM levels (E) and statistical analysis (F) of in vitro suppressive assay of Treg cells purified from spleen in *Ern1*$^{f/f}$ and *Ern1*$^{\Delta Treg}$ mice, and assessed by proliferation of activated CD4^{+} T cells in the presence of various ratios (Tresp : Treg = 16:8, 16:4, 16:2 and 16:1) of Treg cells (n = 3, detected on day 3).

(G) Expression levels of Foxp3 (left panel) and cellular ROS (right panel) in WT splenic Treg cells treated with α-CD3/28 (TCR), thapsigargin (TG) alone or together with 4μ8C for 16-20 h (n = 3).

(H) WB analysis of TRAF2 protein level in WT Treg cells treated with α-CD3/28, thapsigargin (TG) alone or together with KIRA6 for 16-20 h.

(I and J) The FCM plots (I) and analysis (J) of XBP1s in WT Treg cells treated with α-CD3/28, thapsigargin (TG) alone or together with 4μ8C for 16-20 h ($n = 3$).

Data are presented as mean ± SEM of biologically independent samples and represents at least 3 independent experiments, each involving 3 mice per group. NS, not significant, *$p < 0.05$, **$p < 0.01$, ***$p < 0.001$, and ****$p < 0.0001$, by one-way analysis of ANOVA with Tukey's multiple-comparison test (J) and two-tailed Student's t test.

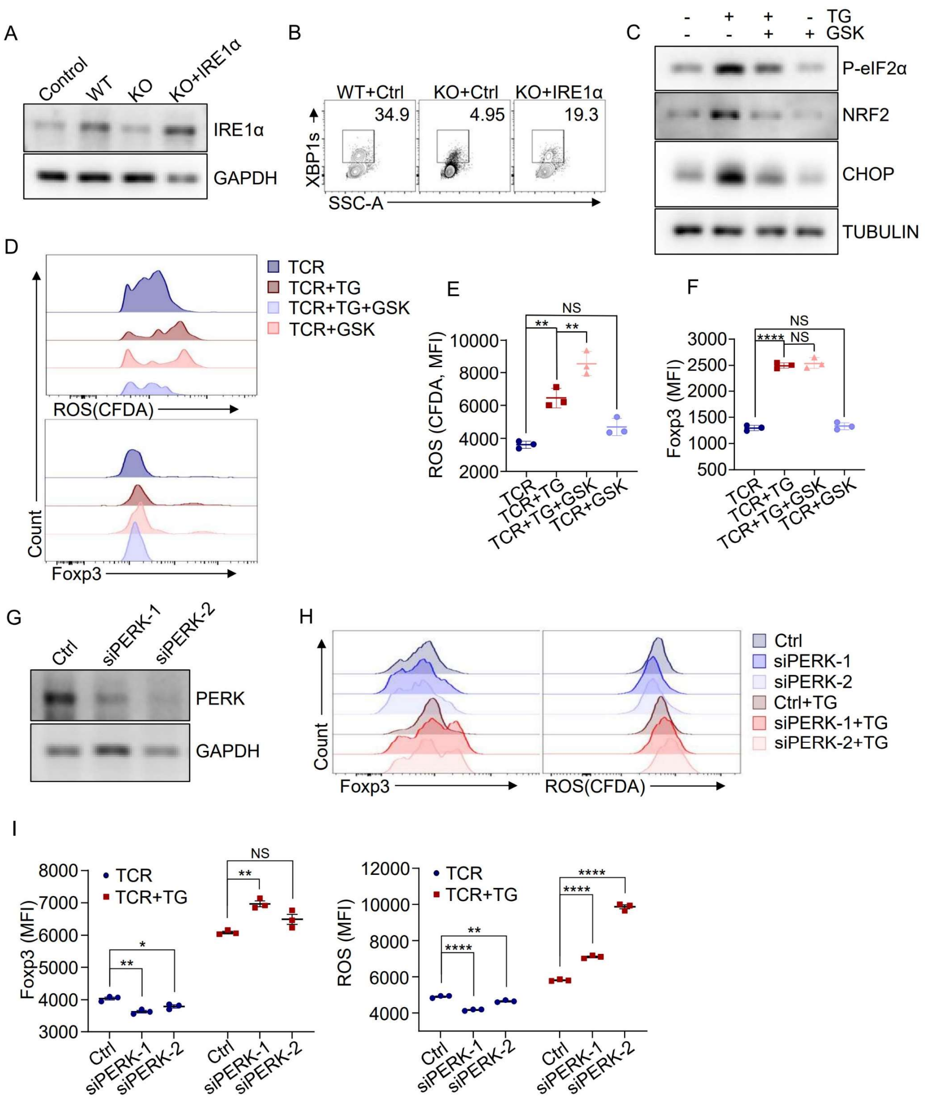

Figure S10. *Tmed4*-deficiency in Treg cells led to lower Foxp3 expression and ROS accumulation in an IRE1α-XBP1 axis dependent manner (Continued)

(A) WB analysis of IRE1α protein levels of iTreg with Vector (Ctrl) or IRE1α-forcible expression.

(B) The FCM plots of XBP1s in WT and *Tmed4*-deficient splenic Treg cells with overexpressing of IRE1α or not (Ctrl). Cells were treated with thapsigargin (TG) for 16-20 h.

(C) WB analysis of PERK pathway proteins in WT splenic Treg cells treated with thapsigargin (TG) alone or together with PERK inhibitor GSK2656157 (GSK) for 16-20 h.

(D-F) The FCM Levels (D) of cellular ROS (D, upper) and Foxp3 (D, below) and their statistical analysis (E and F) in WT splenic Treg cells treated with thapsigargin (TG) or together with PERK inhibitor GSK2656157 (GSK) for 16-20 h (n = 3).

(G) WB analysis of PERK protein levels of Vector (Ctrl) and siPERK-1/siPERK-2 transfected primary iTreg cells.

(H and I) FCM levels (H) and statistical analysis (I) of Foxp3 and ROS levels of Vector (Ctrl) and siPERK-1/siPERK-2 transfected primary iTreg cells (n = 3).

Data are presented as mean ± SEM of biologically independent samples and represents at least 3 independent experiments, each involving 3 mice per group. NS, not significant,

*p < 0.05, **p < 0.01, ***p < 0.001, and ****p < 0.0001, by one-way analysis of ANOVA with Tukey's multiple-comparison test (E-F and I).

Supplementary tables

Table 1：Q-PCR primer sequences

Actin-F	GGCTGTATTCCCCTCCATCG
Actin-R	CCAGTTGGTAACAATGCCATGT
Tmed4-F	GACGGCAAGGTTGTACTGTC
Tmed4-R	GAGAGCCATTCTGGTGGAGT
Bip-F	TCATCGGACGCACTTGGAA
Bip-R	CAACCACCTTGAATGGCAAGA
Chop-F	GTCCCTAGCTTGGCTGACAGA
Chop-R	TGGAGAGCGAGGGCTTTG
Erdj4-F	TAAAAGCCCTGATGCTGAAGC
Erdj4-R	TCCGACTATTGGCATCCGA
Sec61a1-F	CTATTTCCAGGGCTTCCGAGT
Sec61a1-R	AGGTGTTGTACTGGCCTCGGT
Atf4-F	GTGGTTCCCGTGGGTCTCCT
Atf4-R	CTGCTCAGCCCGCTTCTTCT
mt-Cytb-F	TTCATGTCGGACGAGGCTTA
mt-Cytb-R	GTTTATTGGGGATTGAGCGTAG
mt-Nd1-F	CTAGCAGAAACAAACCGGGC
mt-Nd1-R	GTATGGTGGTACTCCCGCTG
mt-Nd4-F	ACAACACACACCTTAGACGCT
mt-Nd4-R	TGTGGATCCGTTCGTAGTTGG
Gclm-F	TGGGCACAGGTAAAACCCAA
Gclm-R	CACCCTGATGCCTAAGCCAA
Hmox-1-F	GAGCAGAACCAGCCTGAACT
Hmox-1-R	AAATCCTGGGGCATGCTGTC
ACTIN-F	CTCTTCCAGCCTTCCTTCCT
ACTIN-R	CAGGGCAGTGATCTCCTTCT
TMED4-F	GGGCTCTACTTCCACATCGG
TMED4-R	TCTGGGTACGATAGTTGCCG
DDIT3-F	CTGCTTCTCTGGCTTGGCTG
DDIT3-R	GCTCTGGGAGGTGCTTGTGA
HSPA5-F	GACGGGCAAAGATGTCAGGA
HSPA5-R	GCCCGTTTGGCCTTTTCTAC
FOXP3-F	GTGGCCCGGATGTGAGAAG
FOXP3-R	GGAGCCCTTGTCGGATGATG

Table 2：Other primer sequences

TMED4-noTag-F	TCTAGAGCCACCATGGCAGGTGTCGGGGCTGG
TMED4-noTag-R	GGATCCTCACACCAGCTTCTTGGCCTCAA
IRE1α-HA-F	GAATTCGCCACCATGCCGGCCCGGCGGCTGCT
IRE1α-HA-R	GCGGCCGCTCAAGCGTAGTCTGGGACGTCGTATGGGT

	AGAGGGCGTCTGGAGTCACTG
siPERK-1-F	GCCACUUUGAACUUCGGUAUA
siPERK-1-R	UAUACCGAAGUUCAAAGUGGC
siPERK-2-F	CCUCUACUGUUCACUCAGAAA
siPERK-2-R	UUUCUGAGUGAACAGUAGAGG

Table 3: Key resources table

**REAGENT or RESOURCE**	**SOURCE**	**IDENTIFIER**
**Antibodies**		
TMED4	Abcam	GR256482-9
TMED4	Proteintech	14141-1-AP
FOXP3	Santa Cruz	sc-166212
ACTIN	Santa Cruz	sc-69879
IRE1α	CST	3294T
IRE1α	Zen Bioscience	251357
PERK	CST	5683T
PERK	Abclonal	A11577
ATF6	proteintech	24169-1-AP
ATF6	Abclonal	A9979
HRD1	proteintech	67488-1
P-IRE1α	Affinity	AF7150
p-eIF2a(Ser51)	CST	3398T
BIP	CST	12721T
BIP	Santa Cruz	sc-13539
CHOP	Santa Cruz	sc-16682
Ub(K48)	CST	8081
TRAF2	CST	9166T
XBP1(u/s)	Abcam	ab37152
XBP1(u/s)	GeneTex	GTX102229
GAPDH	CST	13140
HA	GeneTex	GTX54716
FLAG	Abclonal	AC008
TUBULIN	BD Biosciences	553142
NRF2	CST	12721T
Anti-mouse IgG HRP-linked Antibody	CST	7076s
Anti-rabbit IgG HRP-linked Antibody	CST	7074s
Anti-rat IgG HRP-linked Antibody	CST	7077s
K48 TUBE HF (FLAG)	LifeSensors	LSS-UM-0607-0050
**Flow antibody**		
Fc block CD16/CD32	BD Biosciences	553142
CD4-APC	Biolegend	147311
CD4-FITC	BD Biosciences	557307
CD8-APCCY7	BD Biosciences	557654
CD44-PE	BD Biosciences	553134
CD62L-APC	BD Biosciences	553152

Foxp3-APC	BD Biosciences	560401
CD69-FITC	Biolegend	104506
OX40-APC	Biolegend	119413
ICOS-PE	Biolegend	107706
IFN-γ-APC	BD Biosciences	554413
IL-17-PE	BD Biosciences	559502
CD25-PECY7	BD Biosciences	552880
CD45.2-BV605	Biolegend	109841
CD45.2-APC-cy7	Invitrogen	47-0454-82
CD25-PE	Invitrogen	12-0251-82
CD8a-BV650	Biolegend	100742
CD4-PB	BD Biosciences	558107
GITR-PECY7	Invitrogen	25-5874-80
CTLA-4-APC	eBioscience	17-1522-80
PD-1-FITC	Biolegend	135213
PD-1(CD279)-APC-cy7	Biolegend	135223
Ki67-PE	Invitrogen	12-5698-82
CD45.1-percp-cy5.5	Invitrogen	45-0453-82
Foxp3-e450	Invitrogen	48-5773-82
CD45.2-APC-eFluor™780	Invitrogen	47-0454-82
CD45.2-BV605	Biolegend	109841
CD45RB-PE	Biolegend	151603
IL-17A-percp-cy5.5	Biolegend	506919
IL-17-APC-CY7	BD Biosciences	560821
TNF-α-PE	BD Biosciences	554419
XBP1s-AF647	BD Biosciences	562821
Ly6G- percp-cy5.5	Biolegend	127615
CD11b-FITC	BD Biosciences	557396
Phospho-mTOR-PE-Cy7	Invitrogen	25-9718-41
Phospho-S6	Invitrogen	MA5-16397
hCD45-FITC	Biolegend	304006
hCD4-APC	Biolegend	317416
hCD25-PE	Biolegend	302666
hCD8-PE-Cy7	BD Biosciences	557746
Fixable Viability Stain700	BD Biosciences	564997
Annexin V-FITC/PI Apoptosis Detection Kit	Yeasen	40302ES60
Chemicals and reagents		
Thapsigargin	Sigma	67526–95-8
Tunicamycin	Selleckchem	S7894
Cycloheximide	Selleckchem	S7418
H2DCFDA	Selleckchem	S9687
Mito-SOX™ Red mitochondrial superoxide indicator	Invitrogen	1830251
4μ8C	Selleckchem	S7272
KIRA6	Selleckchem	S8658
GSK2656157	Selleckchem	S7033

NAC	Selleckchem	S1623
Mito Tracker™ Green FM	Invitrogen	M7514
Mito Tracker™ Deep Red FM	Invitrogen	M22426
Sulforaphane	Selleckchem	S5771
Purified Hamster Anti-Mouse CD3e	BD Biosciences	553058
Purified NA/LE Hamster Anti-Mouse CD28	BD Biosciences	553294
Anti-Flag (DYKDDDDK) affinity gel	Selleckchem	B23101
Anti-HA Affinity Beads	Smart-Lifesciences	SA068001
Trizol	Ambion	15596026
Cell proliferation dye eFlour E450	eBioscience	65-0842
DNase I	STEMCELL	9003-98-9
SYBR Green	Yeasen	11201ES03
ECL WB substrate	Tanon	180-5001
Phosphatase inhibitors	Keygen Biotech	KGP602
DMSO	Beyotime	ST038
DCA	Selleck	S8615
Agilent Seahorse XF glycolysis stress test kit	Agilent	103020-100
Agilent Seahorse XF Cell Mito Stress test kit	Agilent	103015-100
RPMI without amino acid	Coolaber science	CM0011
Triton X	Beyotime	ST795
BSA	Yeasen	36101ES60
Percoll	GE-healthcare	17-5446-02
PEI	Polysciences	24765
**Critical commercial assays**		
Hifair II 1st Strand cDNA Sythesis Kit (gDNA digester plus)	Yeasen	11121
PVDF membrane	Immobilon-P	IPVH00010
collagenase IV	Sigma	403a
stimulation cocktail	Invitrogen	00-4975-93
True Nuclear staining kit	Biolegend	73162/73158/73160
