# Supplementary material for: TMED4 facilitates regulatory T cell suppressive function via ROS homeostasis in tumor and autoimmune mouse models
Source: J Clin Invest. 2024 Oct 31;135(1):e179874. doi: 10.1172/JCI179874 (PMC11684806; doi:10.1172/JCI179874)

Full unedited blot for Figure 1M

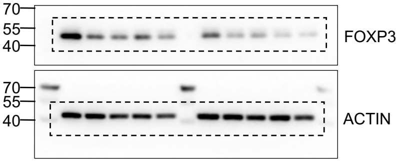

Full unedited blot for Figure 5B(left)

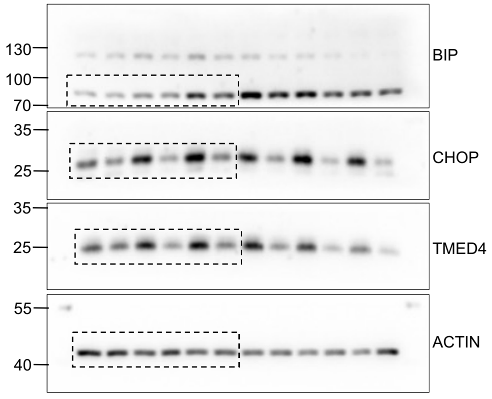

Full unedited blot for Figure 5B(right)

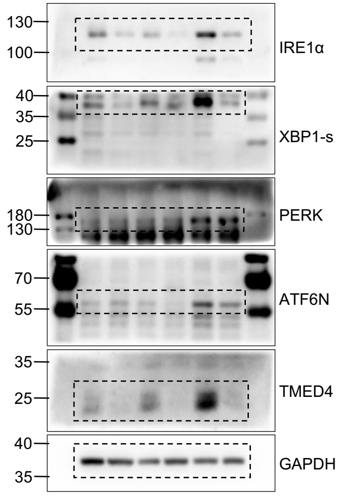

Full unedited blot for Figure 5L

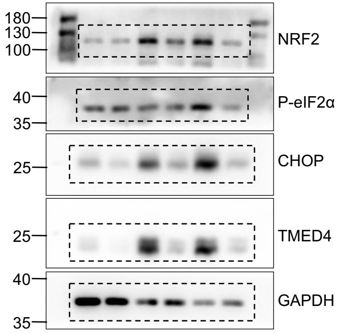

Full unedited blot for Figure 8G

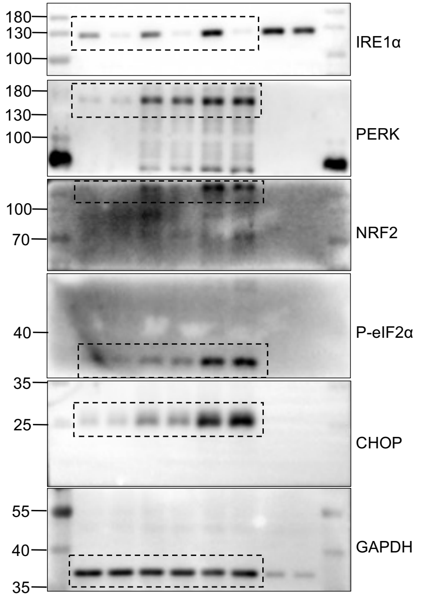

Full unedited blot for Figure 9A

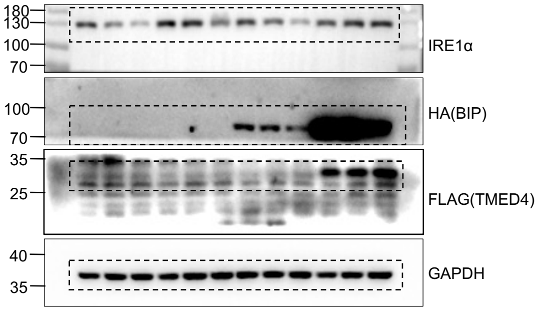

Full unedited blot for Figure 9C

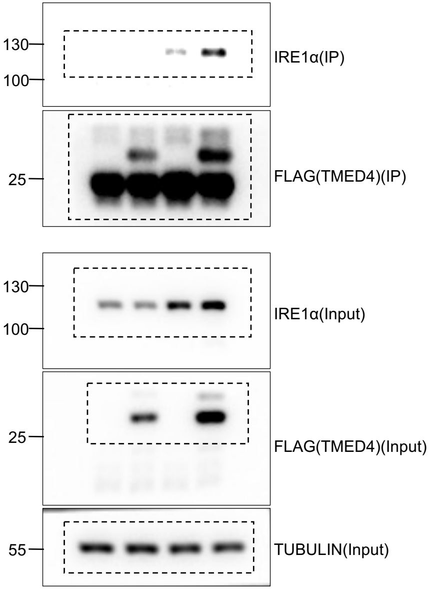

Full unedited blot for Figure 9D

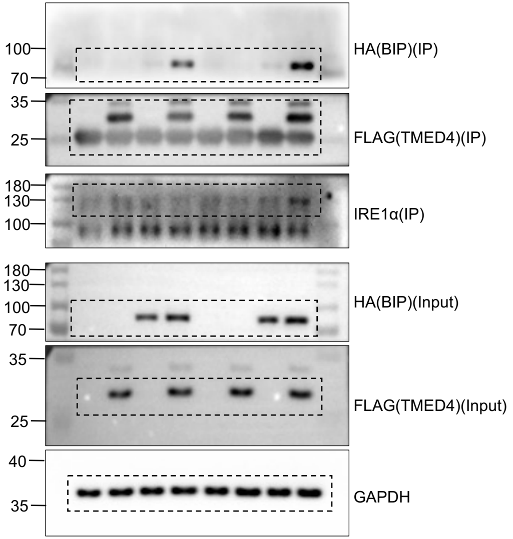

Full unedited blot for Figure 9E

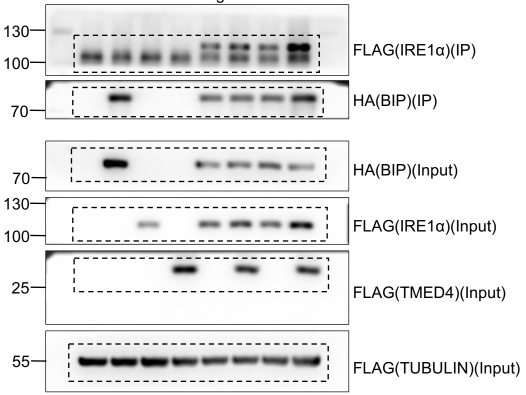

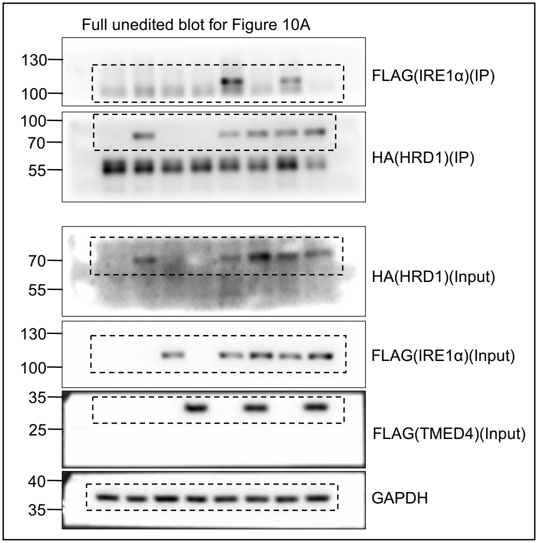
Full unedited blot for Figure 10A
130
100
FLAG(IRE1α)(IP)
100
70
55
HA(HRD1)(IP)
70
55
HA(HRD1)(Input)
130
100
FLAG(IRE1α)(Input)
35
25
FLAG(TMED4)(Input)
40
35
GAPDH

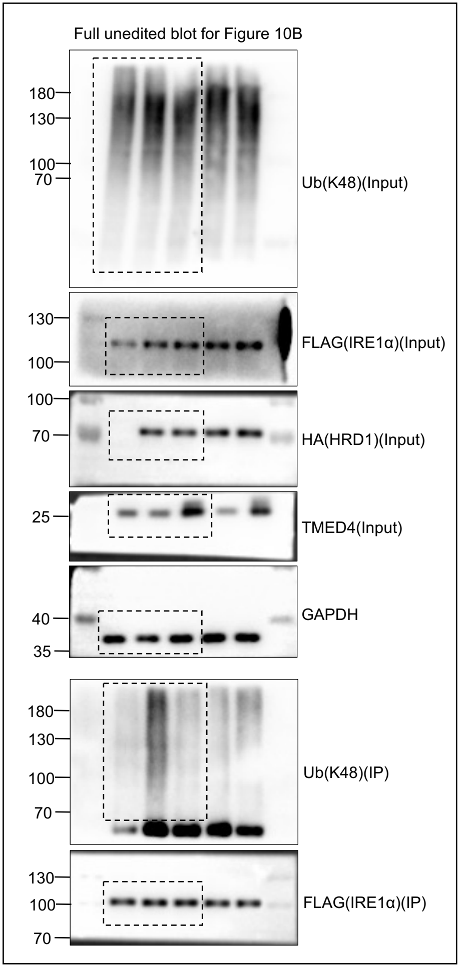
Full unedited blot for Figure 10B
180
130
100
70
Ub(K48)(Input)
130
100
FLAG(IRE1α)(Input)
100
70
HA(HRD1)(Input)
25
TMED4(Input)
40
35
GAPDH
180
130
100
70
Ub(K48)(IP)
130
100
70
FLAG(IRE1α)(IP)

Full unedited blot for Figure 10C

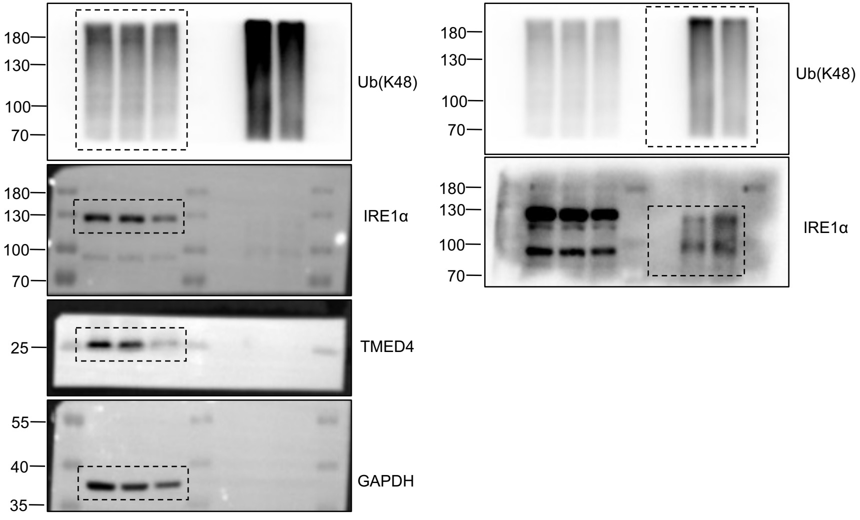

Full unedited blot for Figure S2A

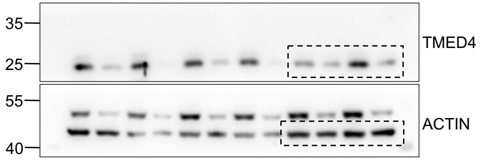

Full unedited blot for Figure S9H

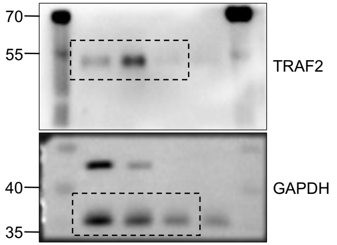

Full unedited blot for Figure S6A

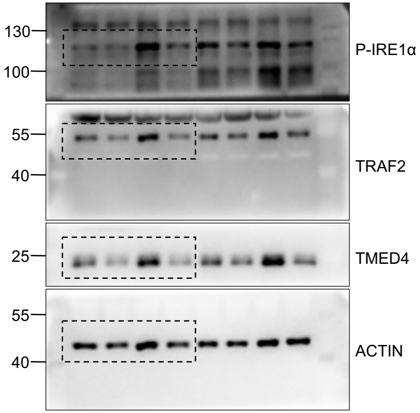

Full unedited blot for Figure S7D

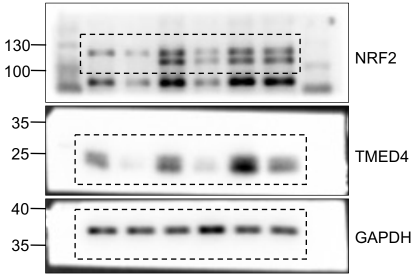

Full unedited blot for Figure S10A

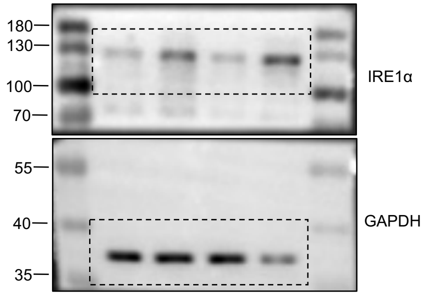

Full unedited blot for Figure S10G

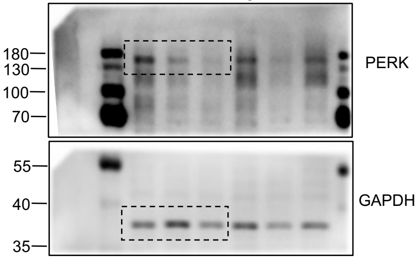

Full unedited blot for Figure S10C

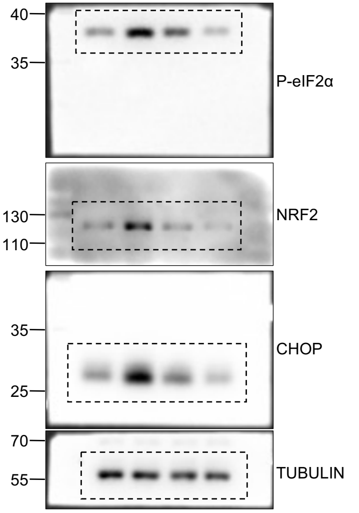

Supplement: Unedited blot and gel images [file jci-135-179874-s012.pdf]
